# An assessment of whether long-term global changes in waves and storm surges have impacted global coastlines

**DOI:** 10.1038/s41598-023-38729-y

**Published:** 2023-07-17

**Authors:** Mandana Ghanavati, Ian Young, Ebru Kirezci, Roshanka Ranasinghe, Trang Minh Duong, Arjen P. Luijendijk

**Affiliations:** 1 Department of Infrastructure Engineering, University of Melbourne, Melbourne, VIC 3010 Australia; 2 Department of Coastal and Urban Risk & Resilience, IHE Delft Institute for Water Education, P.O. Box 3015, 2601 DA Delft, The Netherlands; 3 Resilient Ports and Coasts, Deltares, P.O. Box 177, 2600 MH Delft, The Netherlands; 4 Water Engineering and Management, Faculty of Engineering Technology, University of Twente, P.O. Box 217, 7500 AE Enschede, The Netherlands; 5 Department of Hydraulic Engineering, Faculty of Civil Engineering and Geosciences, Delft University of Technology, Delft, The Netherlands

**Keywords:** Physical oceanography, Environmental impact

## Abstract

A common inference in research studies of observed and projected changes in global ocean wave height and storm surge, is that such changes are potentially important for long-term coastal management. Despite numerous studies of the impacts of anthropogenic climate change on trends in global wind and waves, a clear link to impacts on sandy coastlines, at global scale, is yet to be demonstrated. This study presents a first-pass assessment of the potential link between historical trends in global wave and storm surge values and recession/progradation rates of sandy coastlines since the 1980s. Global datasets of waves, surge and shoreline change rate are used for this purpose. Over the past 30 + years, we show that there have been clear changes in waves and storm surge at global scale. The data, however, does not show an unequivocal linkage between trends in wave and storm surge climate and sandy shoreline recession/progradation. We conclude that these long-term changes in oceanographic parameters may still be too small to have a measurable impact on shoreline recession/progradation and that primary drivers such as ambient imbalances in the coastal sediment budget may be masking any such linkages.

## Introduction

Being home to between 10 to 13% of the global population, the low elevation coastal zone (LECZ: coastal land zone at less than 10 m above mean sea level) is arguably the most inhabited land zone in the world^1^. Close to one third (31%) of the global coastline is classified as sandy coastlines^2^, which are very dynamic systems, changing at a multitude of spatio-temporal scales in response to local oceanographic drivers such as waves, tides, storm surges and sea level rise, as well as in response to terrestrial drivers (e.g. fluvial sediment loads) and human activities (e.g. engineering structures, land reclamations). Climate change, and associated mean sea level rise, is projected to result in widespread coastal recession along sandy coasts through the twenty-first century^3^, potentially disrupting lives and leading to massive socio-economic losses^4,5^. Therefore, adequately understanding how climate change may affect sandy coastlines is of great importance. Although, some first-pass projections of how sandy shorelines may change over the twenty-first century are now available^4,6,7^, these estimates have been obtained using simplistic models that facilitated global scale assessments. Currently available data sets of sandy shoreline change (recession/progradation) and associated oceanographic drivers (sea level rise, waves, storm surges) over the last few decades do, however, lend themselves to a more detailed historical analysis of the potential forcing-response linkages between oceanographic drivers of sandy shoreline change, at global scale. Such an analysis constitutes the focus of the present study.

In recent years, significant research has focused on studies of historical long-term variability of ocean wave climate, including trends of wave height and storm frequency both at the global and regional scale^8–16^. Several studies have also focused on regional and global trends in storm surge level, intensity and frequency^17–23^. The global climate and hence winds, waves, and storm surge change at multiple scales. Muti-decadal oscillations (e.g. El Niño) are known to influence wind and wave conditions and have also been shown to impact coastal erosion^24–26^. The above studies also show that long-term anthropogenic changes in climate give rise to trends in winds and waves. It is often claimed that these long-term changes in ocean wave climate and extremes may potentially have impacts on coastal ecosystems, sandy beach recession, evolution and beach alignment^5,16,27–32^. If this is the case, future changes in wave and storm surge conditions, could be projected to impact beaches. Despite the many claims of a potential link between changing wave and surge conditions and sandy shoreline change, to our knowledge, no study has ever been undertaken to determine whether there is, in fact, a measurable link between historical sandy shoreline recession/progradation trends and wind waves and storm surge trends, at global scale.

In the following, we use the terminology recession/progradation to respectively describe the shoreward/seaward movement of the mean beach location averaged over a long period of time (here 3 decades). This can be contrasted with erosion/accretion which are associated with short-term changes in shoreline position as a result of individual storms or medium-term changes in weather patterns (e.g. El Niño)^24^.

In this paper, we combine data from a variety of global and regional datasets to examine trends in recent decades in wave and surge parameters at coastal locations, globally. These are compared with satellite derived recession/progradation rates at sandy beaches over the same period. It should be noted that the purpose of this study is to investigate if there is a clear linkage between long term trends in wave and storm surge conditions and rates of recession/progradation over three decades, rather than shorter-term shoreline changes. Global data for wave parameters (wave energy flux, wave height, wave period, wave direction and number of extreme wave events) and storm surge (surge level and number of storm surge events) at coastal locations are considered, using available global datasets. Also, to add more detail and to validate the use of global-scale deep water wave model data, a high-resolution regional dataset of wave conditions for southern Australia is used. The historical recession/progradation of sandy beaches is determined using two satellite derived global datasets. A regional recession/progradation dataset for the Australian coastline is used for further validation. Finally, potential linkages between long-term changes in oceanic waves, storm surge and sandy shoreline recession/progradation are investigated at global-scale.

## Results

All the datasets used in this study are described in detail in the “Methods” section. In summary, historical values of parameters were obtained over the period from 1984 to 2014 for surge data and 1984–2016 for waves and shoreline recession/progradation data. As no global, high resolution coastal (i.e. nearshore) database of wave conditions is available, the deep-water data of the Liu et al.^33^ hindcast were used. The spatial and temporal resolution of this hindcast is 0.25° and 3-h, respectively. This Liu et al. hindcast has been extensively validated globally against buoy and satellite data^15,33,35^.

Storm surge data were obtained from the GTSR^36^ (global tide and surge reanalysis) dataset. This is a global hindcast with a temporal resolution of 10 min and a spatial resolution varying from 50 km in deep water to 5 km in nearshore regions. These surge data have been extensively validated at tide gauge locations globally^36,37^. Two global satellite-derived coastal recession/progradation datasets were unitized, Luijendijk et al.^2^ (500 m along-coast resolution) and Mentaschi et al.^38^ (250 m along-coast resolution).

Each of the three data types (waves, storm surge and recession/progradation) need to be assigned to coastal locations to facilitate further analysis. By their nature, the recession/progradation data are defined at specific sandy beach locations. The GTSR data (surge) are defined on an irregular grid. These surge data have, however, been resampled^20^ and provided at 9866 irregularly spaced coastal locations defined by the Dynamic Interactive Vulnerability Assessment database (DIVA)^37,39^. The wave data are defined at deep-water grid locations on a 0.25° resolution grid. Therefore, grid points for the deep water wave datasets closest to the shoreline were taken to be representative of sandy beach locations. Note that the two shoreline change datasets are not spatially co-located nor are the surge data, defined at DIVA locations and the wave data, defined on its regular grid. Hence, although all datasets are associated with coastal locations, none are co-located and each has a different number of shoreline points.

As noted above, the Liu et al. wave data are deep-water, and no nearshore transformation of waves has been considered. The nearshore wave conditions potentially driving shoreline recession/progradation are likely to be different from these deep-water wave conditions. However, our interest here is in large spatial scale trends in these quantities. We assume that if there is, for instance, a positive trend in a wave quantity offshore, a trend of the same sign will also occur for nearshore waves. Although there will be specific local sites for which this assumption may not hold, we believe that it is a reasonable assumption for such a global-scale analysis, given that no global nearshore wave data are available (see “Methods” for a more detailed discussion). To investigate the validity of this assumption, a comparison with a regional, high-resolution hindcast dataset^40^ for the southern Australian coast was undertaken. This comparison confirmed that deep-water trends from the global datasets were consistent with nearshore trends (i.e. the signs were the same, although nearshore trends were usually slightly smaller in magnitude—see “Limitations and validation” section and Supplementary Fig. S21).

As noted above, global sandy shoreline change rate information was obtained from two independent beach recession/progradation datasets: a modified Luijendijk et al.^2^ dataset (henceforth referred to as the *Delft* dataset, the location of the originators of the data), ($\Delta C_{D}$) and the Mentaschi et al. dataset^38^ (henceforth referred to as the *JRC* dataset, as it was developed at the Joint Research Centre of the European Commission), ($\Delta C_{JRC}$) (see “Methods” for details). Both datasets are obtained from satellite imagery of coastlines. For validation purposes, a high-resolution regional recession/progradation dataset for Australia, compiled by *Geoscience Australia* ($\Delta C_{GA}$)^41^ over the period 1988–2019 was also used. This dataset has a resolution of 30 m along the Australian coastline.

### Trend analysis of wave parameters

The following wave parameters were extracted from the Liu et al. wave dataset: significant wave height, $H_{s}$; mean wave period, $T_{m}$ and mean wave direction, $\theta_{m}$. These quantities were used to calculate a number of wave-related parameters, including, wave energy flux ($C_{g} E = \rho g^{2} H_{s}^{2} T_{m} /(64\pi )$), number of extreme wave height events, ($N_{{H_{s}^{95} }}$—number of $H_{s}$ events above 95th percentile with at least a 48 h separation) and annual values of the high percentiles (95th, 98th, 99th, 99.5th) (see “Methods”).

To investigate the global distribution of trends in wave parameters, annual values of the quantities, $C_{g} E$, $H_{s}$, $T_{m}$, $\theta_{m}$ and $N_{{H_{s}^{95} }}$ were calculated and the trends over the period 1984–2016 determined using both Sen’s slope estimation^42^ and linear regression. This process was also repeated for the upper percentiles, as above, with the 95th percentile subsequently being adopted as a proxy for extreme wave conditions (see “Methods”). Note that throughout this study, use of the symbol, $\Delta$ indicates a trend (e.g. $\Delta H_{s}$ is the trend in significant wave height).

Numerous previous studies have examined global trends in significant wave height from both satellite altimeter and model data^8,13,16,27^. These results consistently show that over recent decades there has been a statistically significant increase in $H_{s}$ across the Southern Ocean of approximately 0.5 cm/year. The swell emanating from the Southern Ocean means that these positive trends are also present across much of the Southern Hemisphere. However, the North Atlantic and North Pacific show much smaller changes which are generally not statistically significant and with no clear sign (i.e. increasing or decreasing).

**Figure 1:**
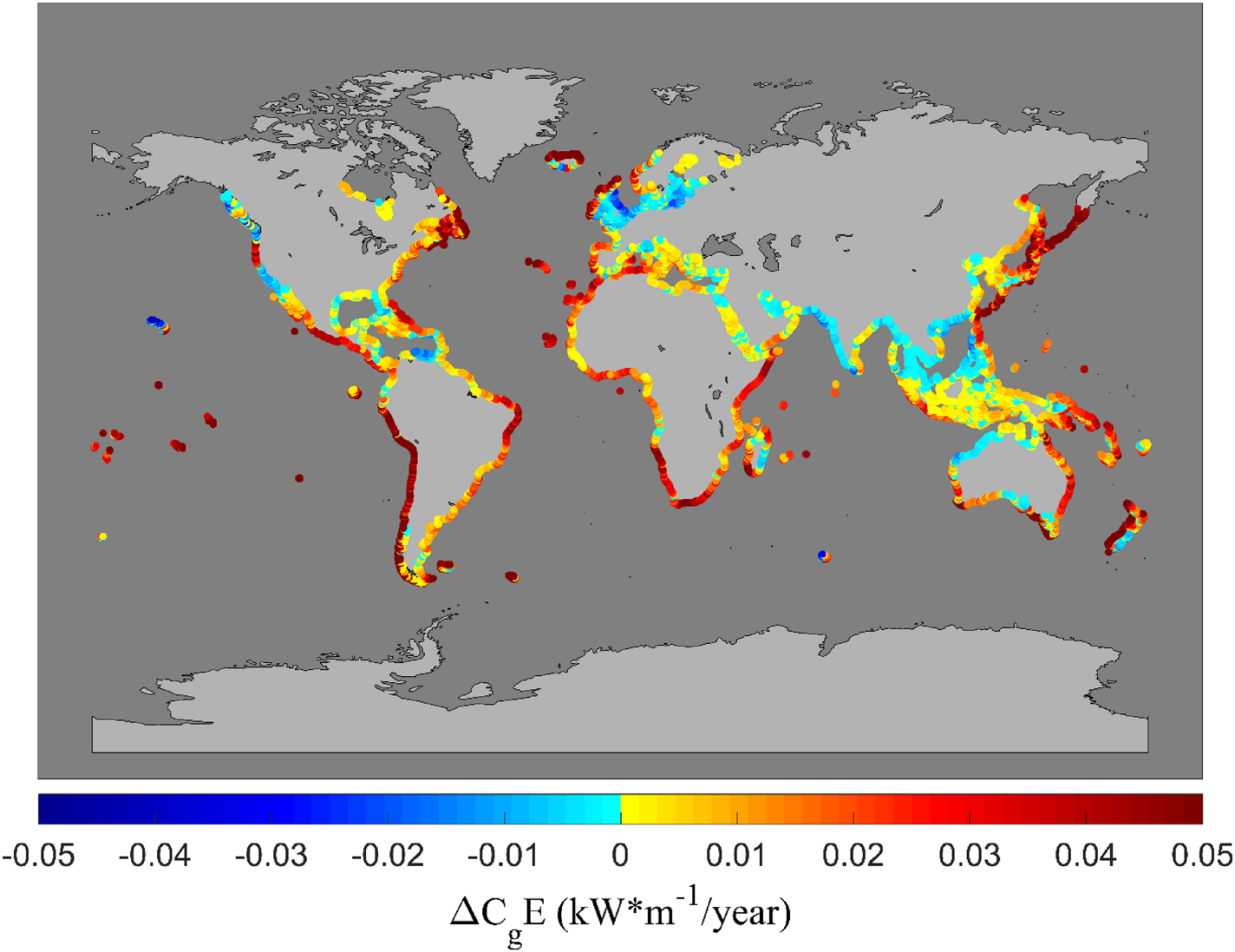
Global distribution of trend in annual average values of wave energy flux, $\Delta C_{g} E$(kWm^−1^/year) at sandy beach locations for the period of 1984–2016 from the Liu et al.^33^ dataset [Map generated using Matlab Mapping toolbox].

Figure 1 shows the Liu et al. values of $\Delta C_{g} E$ at coastal locations (the corresponding values of $\Delta H_{s}$ are shown in Figure S1). The wave energy flux, $C_{g} E$, is often seen as a driver of beach erosion and evolution as it includes both the impacts of $H_{s}$ and $T_{m}$. This figure shows relatively large ($\Delta C_{g} E \approx$ 0.05 kWm^−1^/year) trends along western coasts, particularly for South America, southern Africa, Western Australia and New Zealand. This is a result of the positive trends in $H_{s}$ across the Southern Ocean and the increases in $T_{m}$ in the swell “pools” along the western coasts of these continents. There are also relatively strong positive trends along the coasts of Japan, as a result of increasing values of wave energy flux in the western portions of the North Pacific. The west coast of North America and northern Europe show more variable trends in wave energy flux.

The global distribution of trends in the annual values of the other wave parameters are shown in the Supplementary Material—extreme wave height ($\Delta H_{s}^{95}$) (95th percentile), Supplementary Fig. S3; Number of extreme wave height events ($\Delta N_{{H_{s}^{95} }}$), Supplementary Fig. S4; mean wave period ($\Delta T_{m}$), Supplementary Fig. S5 and mean wave direction ($\Delta \theta_{m}$), Supplementary Fig. S6. Consistent with several previous studies from both model hindcast and satellite altimeter data, Fig. 1 and Supplementary Figs. S1–S6 generally show a global increase in all these wave properties over the historical period. These increases are larger in the Southern Ocean, which results in enhanced swell across much of the Pacific, Indian and South Atlantic Oceans. Trends in the northern hemisphere are smaller than in the southern hemisphere but still generally positive. Areas where there are clear negative trends include: the coasts of western Europe, the north-west coast of Australia (where the angle of the shoreline protects this coastline from Southern Ocean swell) and the coasts of Asia (where model resolution will not adequately capture tropical cyclone activity).

Table 1 shows statistical summary information for these quantities for various regions of the world. The Liu et al. dataset indicates that 73% (Table 1) of world coastlines show an increasing trend in $C_{g} E$, 74% show an increasing trend in $H_{s}$, 71% show an increasing trend in $H_{s}^{95}$, 61% show an increasing trend in $N_{{H_{s}^{95} }}$ and 76% in $T_{m}$.

**Table 1:** Percentages of coastlines showing positive trends in wave and surge parameters, and of sandy shorelines showing recession for world and continent coastline. For $\Delta C_{D}$ only absolute values between 1 and 5 m/year considered, with values outside these limits being excluded from the percentage calculations.

		Sandy shoreline recession $\Delta C_{D}$	Increasing trend of $\Delta C_{g} E$	Increasing trend of $\Delta H_{s}$	Increasing trend of $\Delta H_{s}^{95}$	Increasing trend of $\Delta N_{{H_{s}^{95} }}$	Increasing trend of $\Delta T_{m}$	Increasing trend of $\Delta \eta^{95}$	Increasing trend of $\Delta N_{\eta 95}$
World coastline		47%	73%	74%	71%	61%	76%	70%	42%
South America coastline	West	42%	93%	97%	93%	39%	75%	63%	46%
	East	49%	86%	77%	85%	68%	82%	86%	35%
North America coastline	West	62%	68%	82%	76%	55%	53%	86%	25%
	East	50%	87%	82%	81%	63%	87%	84%	54%
Africa coastline	West	56%	92%	89%	79%	64%	80%	49%	45%
	East	46%	99%	100%	93%	78%	99%	36%	36%
Australia coastline	West	46%	35%	20%	73%	16%	89%	79%	28%
	East	47%	99%	99%	98%	56%	99%	36%	29%
Western Europe	West	48%	63%	50%	26%	63%	69%	51%	68%

These clear indications of globally increasing wave property trends have been the basis for speculation as to the potential impacts on coastlines^28–30,43–47^.

### Trend analysis of surge parameters

In a similar fashion to wave conditions, trends in storm surge were investigated from the GTSR dataset. GTSR provides 10 min values of water surface elevation, measured about the mean sea level (i.e. no astronomical tides included). To represent storm surge, here we consider the 95th percentile values of the sea surface elevation ($\eta^{95}$). The trend in the 95th percentile water level (surge), $\Delta \eta^{95}$ was determined using annual values, as for wave conditions. This process was also repeated using 98th, 99th and 99.5th percentiles, yielding similar results (see “Methods”). Also, the number of the storm surge events per year, $N_{\eta 95}$ was determined by counting the number of extreme events above the 95th percentile, with a minimum 48 h separation (see “Methods”) and the trend in number of storm surge events per year, $\Delta N_{\eta 95}$,was also determined.

**Figure 2:**
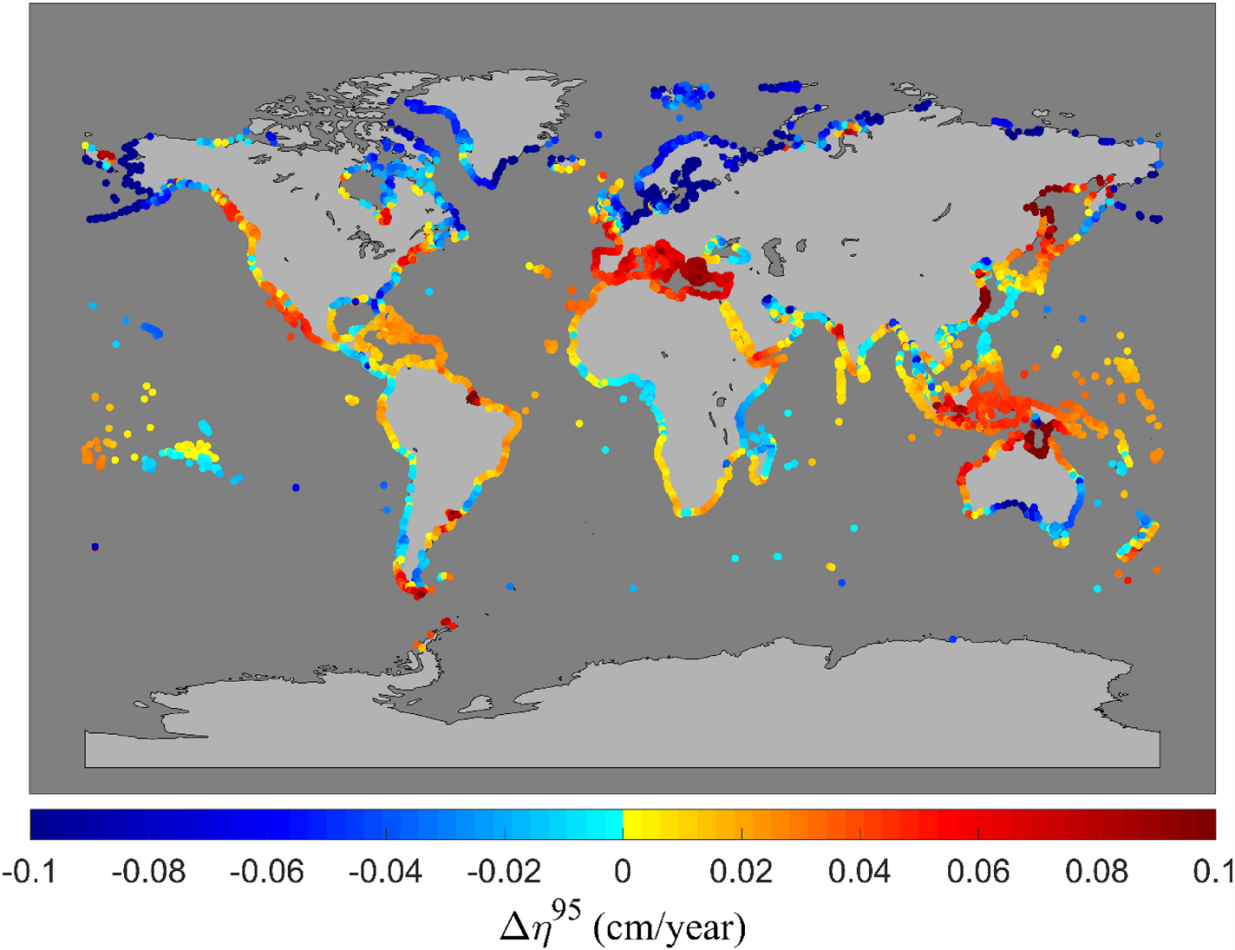
Global distribution of trend in the magnitude of storm surge, $\Delta \eta^{95}$(95th percentile of water level) (cm/year) over the period of 1984–2014 at DIVA locations. Note values in the Arctic can be smaller than the lower limits of the colour bar (up to − 0.3 cm/year) [Map generated using Matlab Mapping toolbox].

Figure 2 shows the global distribution of the trend in storm surge level (95th percentile) $\Delta \eta^{95}$ for the period of 1984–2014 at DIVA locations. It should be noted that the GTSR spatial resolution is too coarse to adequately resolve tropical cyclones. As a result, surge values and subsequent trends are likely to be unreliable in tropical and sub-tropical regions^36^. We are not aware of previous global estimates of storm surge trends from model data. Although, Tadesse et al.^23^ did determine storm surge trends at tide gauge locations concentrated in western Europe, North America, Japan and southern Australia. Their findings^23^ (see their Fig. 5) are generally consistent with the present results. The results for surge show much greater spatial variability than for significant wave height. However, there is still spatial coherence in the data with many extended coastal regions showing consistent values of $\Delta \eta^{95}$.

Coastal areas at higher latitudes (around Arctic) show strong negative trends of surge level (95th percentile) up to 0.3 cm/year (note the color bar is capped at 0.1 cm/year for plotting clarity). The south and south-east coasts of Australia also indicate consistent negative trends. The Mediterranean, north coast of Australia (possibly impacted by tropical cyclones and limited model resolution), south coast of South America, west coast of North America and coastal locations along the east coast of Asia (tropical cyclones) show consistent positive trends in storm surge of up to 0.1 cm/year. Based on the results shown in Table 1, 70% of global coastlines show increasing trends in storm surge levels. Although the results are not as clear as for wave conditions, both datasets showing increasing values is not unexpected, as both global models are driven by consistent wind fields (Liu et al. wave hindcast forced with ERA5 winds^34^ and the GTSR surge results forced with the earlier ERA-Interim winds^48^). Thus, one would expect that increases in global winds (and extreme winds)^16,49,50^ would result in global increases in both waves and storm surge. Waves are possibly more consistent across the globe since swell generated in the Southern Ocean propagates into most oceanic basins thus resulting in more consistent globally positive trends. Obviously, storm surges do not propagate large distances in this same manner and are also impacted by pressure differences, in addition to winds.

Supplementary Figure S7 shows the global distribution of trend in number of storm surge events ($\Delta N_{\eta 95}$) over the period 1984–2014 at DIVA locations. In contrast to the trend in the magnitude of storm surge ($\Delta \eta^{95}$), the trend in the number of storm surge events do not show strong spatial coherence, although individual locations show values up to $\pm 0.3$ events/year. Table 1 also shows that globally, only 42% of coastlines show an increase in the number of storm surge events. That is, increases and decreases are approximately equally represented. This result may, however, be influenced by tropical cyclone regions where the GTSR resolution is inadequate.

### Global shoreline recession and progradation

Two independent beach recession/progradation datasets were used in this study. This allowed a comparison between these datasets, as well as comparisons with the wave and storm surge trend data. These global datasets of shoreline change are detailed in Luijendijk et al.^2^ ($\Delta C_{D}$) and Mentaschi et al.^38^ ($\Delta C_{JRC}$) (see “Methods”). Both datasets are obtained from satellite observation of the coastline. Determining changes in beach locations from such data is computationally complex, involving the requirements of determining the water-land interface under a variety of lighting conditions, the magnitude of both astronomical and meteorological tides at the time of the observation and averaging over sufficient images to remove the impacts of swash or wave runup. For further detailed study and verification, a regional dataset from *Geoscience Australia*^51^ ($\Delta C_{GA}$), with much finer resolution, was also used.

The spatial resolution (along the coast) of the *Delft* dataset is 500 m and the data are classified as either sandy or non-sandy beaches. For further analysis, data points corresponding to sandy locations were extracted from an updated version of this dataset (that excludes some contentious sandy locations which were present in the original data set). Shoreline change rates ($\Delta C_{D}$ (m/year)) were determined at all these sandy locations over the period 1984–2016.

The *JRC*^38^ dataset ($\Delta C_{JRC}$) is also global, with an along coast spatial resolution of 250 m. Values of $\Delta C_{JRC}$ are available over the period 1984–2015 (see “Methods”). However, the data period at each location differs with commencing dates varying between 1984 and 1988. This dataset also does not differentiate between sandy and non-sandy beaches. Based on the *Delft* dataset, there can be significant differences in recession/progradation rates depending on beach type. Therefore, we took the $\Delta C_{D}$ sandy location dataset as a reference. For each $\Delta C_{JRC}$ location, the closest $\Delta C_{D}$ location was taken as an indication for the beach type, provided these points were within 1 km of each other (see “Methods”). As the two datasets are not co-located, a point-by-point comparison is not possible. Also, it should be noted that the $\Delta C_{JRC}$ dataset has a much smoother (stylized) coastline representation than the $\Delta C_{D}$ dataset. A similar approach was used to determine sandy beach locations in the high resolution *Geoscience Australia* ($\Delta C_{GA}$)^51^ dataset used for validation purposes.

In order to investigate the consistency of the sandy shoreline change datasets, we first compare the values of $\Delta C_{JRC}$ and $\Delta C_{D}$. Although the present study is global scale, we speculate that continental coastlines will be affected differently by potential changes in wave and storm surge conditions (e.g. west coasts vs. east coasts). We have therefore investigated results for 9 coastlines: west and east coasts of South America, North America, Africa and Australia and Western Europe. Note that Asia has not been considered, as much of this area will potentially be impacted by tropical cyclone activity, which we cannot resolve with the available datasets.

**Figure 3:**
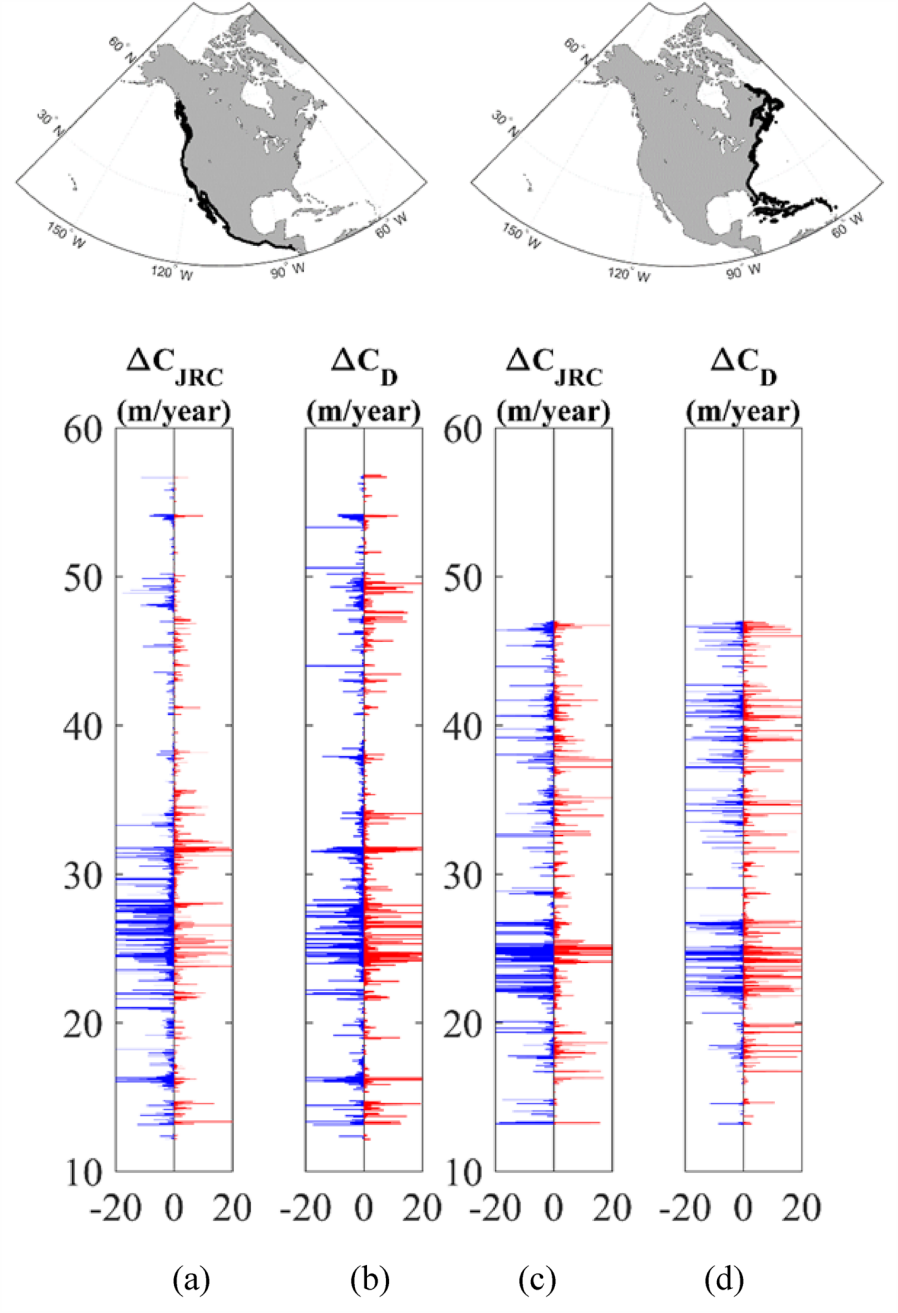
Latitudinal distribution of shoreline change rate of sandy shorelines for the west coast of North America for (**a**) JRC^38^ ($\Delta C_{JRC}$) and (**b**) Delft^2^ ($\Delta C_{D}$) datasets, respectively. Similarly, (**c**) and (**d**) show latitudinal distribution of shoreline change rate of sandy shorelines for the east coast of North America for JRC^38^ ($\Delta C_{JRC}$) and Delft^2^ ($\Delta C_{D}$) datasets, respectively. Recession shown in blue and progradation in red. Rates capped at maximum values of $\pm$ 20 m/year. The inserts at the top show the continent of North America highlighting the coastline regions considered [Map generated using Matlab Mapping toolbox].

Figure 3 shows bar plots for the west and east coast of North America (as an example) indicating the latitudinal distribution of shoreline change rate of sandy shorelines from both global datasets. Blue bars indicate negative values (recession) and red represents positive values (progradation). See also, Supplementary Figs. S8–S11 (Supplementary Material) showing similar comparisons for South America, Africa, Australia and Western Europe, respectively.

A number of points should be considered in interpreting these shoreline recession/progradation data.

Sandy beaches are dynamic systems retreating and progradating over time. A system in equilibrium would, however, show little net recession/progradation over an extended period (decades). As we have data over a long period of time, non-zero values indicate beaches which are not in equilibrium.

Another point to note in interpreting Fig. 3 and Supplementary S8–S11 is that the $\Delta C_{JRC}$ and $\Delta C_{D}$ values are not, necessarily at the same locations, as the coastal points are not co-located. The spatial variability of the data precludes interpolation to common locations and, hence, a point-by-point analysis is not possible. Rather a comparison must qualitatively determine if similar recession/progradation signatures are visible in the dataset along regions of the coast.

The shoreline change measured by these datasets will be a result of a range of physical processes. These will include the available sediment budget in each coastal compartment and the impacts of waves and storm surge on this sediment budget, together with possible sea level change, as well as fluvial process at the mouths of rivers and estuaries. In addition, human interventions such as beach restoration and sand nourishments will also be captured in these data. Figure 3 and Supplementary Figs. S8–S11 have axes of the range $\pm$ 20 m/year, with the larger values almost certainly the result of fluvial and human-induced processes. These plots provide the reader an overview of the magnitudes of the shoreline changes observed. In subsequent analyses we limit data to a range of $\pm$ 5 m/year to exclude major impacts from human-induced activities.

Although there are obvious differences between the datasets, Fig. 3 also shows many similarities. The first notable feature of both datasets are the “bursts” of relatively high values of grouped recession and progradation. That is, when high recession occurs, it is often coupled with high progradation at neighboring beaches. Examples of this which are consistent across both datasets include: east coast North America (Fig. 3c,d) at approximately + 22° to + 27° and west coast of North America (Fig. 3a,b) at approximately + 20° to + 30° and + 17°. Comparisons of the datasets for other global coastlines are shown in the Supplementary Material.

Generally, across all these comparisons, the two datasets indicate shoreline change trends of comparable magnitudes. Noting that the points are not co-located, we believe the similarities between the datasets provide confidence that a shoreline change signal is robustly detected in the underlying satellite data ($\Delta C_{JRC}$ and $\Delta C_{D}$). For subsequent analysis we have adopted the *Delft*^2^ dataset ($\Delta C_{D}$), as it provides a direct indication of the beach type (sandy or non-sandy).

### Relationship between global recession/progradation rates and changes in waves and storm surge properties

**Figure 4:**
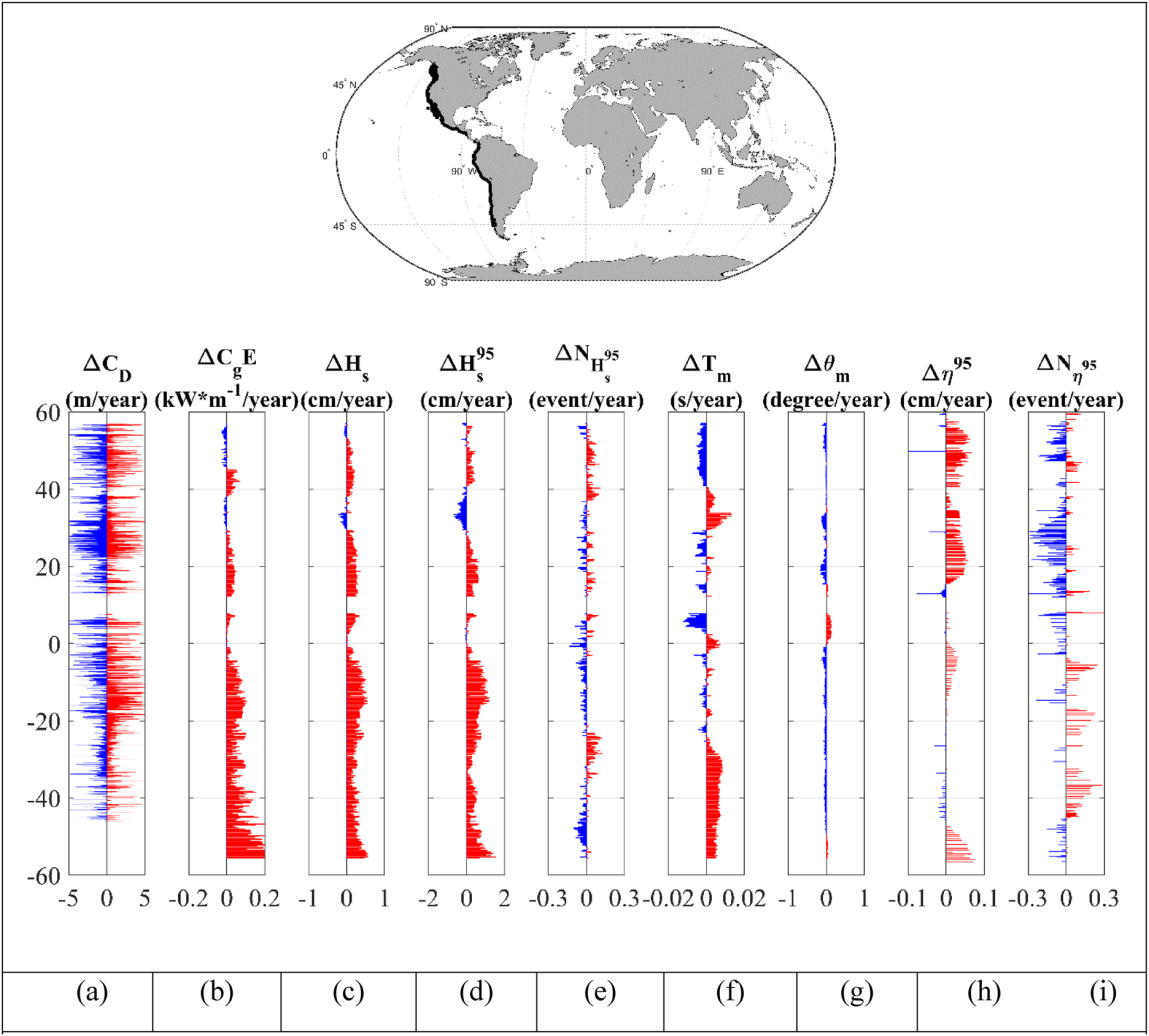
Latitudinal distribution of: (**a**) shoreline recession/progradation,$\Delta C_{D}$ for sandy beaches (blue is recession and red is progradation), (**b**) trend in wave energy flux ($\Delta C_{g} E$), (**c**) trend in significant wave height ($\Delta H_{s}$), (**d**) trend in 95th percentile significant wave height ($\Delta H_{s}^{95}$), (**e**) trend in number of extreme wave events above the 95th percentile ($\Delta N_{{H_{s}^{95} }}$), (**f**) trend in mean wave period ($\Delta T_{m}$), (**g**) trend in mean wave direction ($\Delta \theta_{m}$) (clockwise positive), (**h**) trend in storm surge ($\Delta \eta^{95}$) and (**i**) trend in number of storm surges events ($\Delta N_{\eta 95}$) for the west coast of North and South America. Increasing trends shown in red and decreasing trend in blue. Wave parameters are provided from the Liu et al.^33^ dataset. The insert at the top shows the continents of North and South America highlighting the coastline region considered [Map generated using Matlab Mapping toolbox].

**Figure 5:**
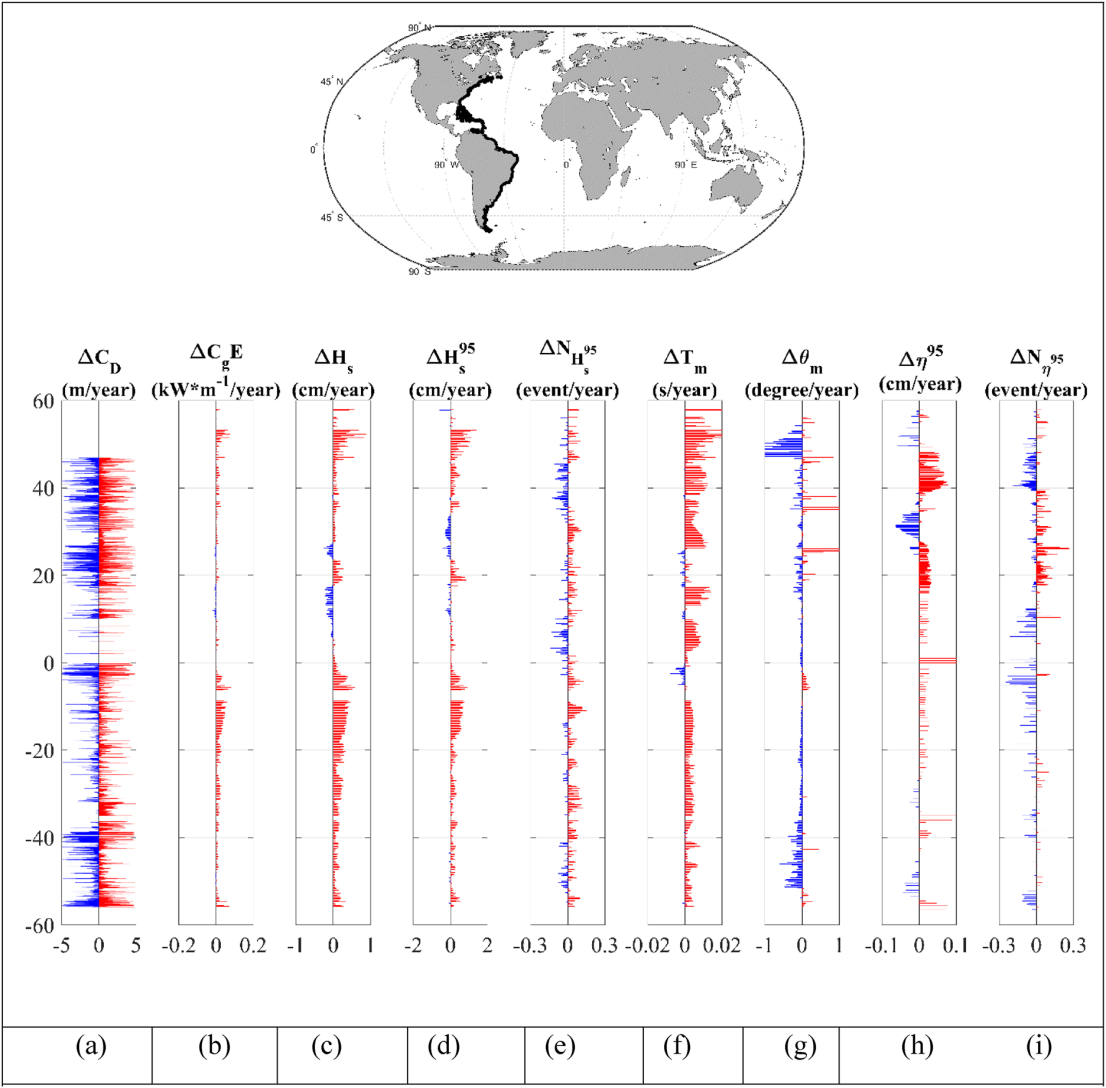
As for Fig. 4 but for the east coast of North and South America.

For a more detailed analysis and better understanding of the relative contributions of wave and storm surge forcing to shoreline change, we again considered the latitudinal distribution of the 9 regions: west and east coasts of North and South America combined (Figs. 4 and 5), North America (Supplementary Figs. S12 and S13), South America (Supplementary Figs. S14 and S15), Africa (Supplementary Figs. S16 and S17), Australia (Supplementary Figs. S18 and S19) and Western Europe (Supplementary Fig. S20). Statistics for each coastline are also shown in Table 1. Note that Asia has not been included in the analysis, as neither the wave nor surge models have a resolution which can adequately capture tropical cyclone activity^36,37^.

Each of these figures shows the latitudinal distribution of the shoreline recession/progradation,$\Delta C_{D}$, together with changes in the physical parameters that may have an impact on this shoreline change—wave energy flux ($\Delta C_{g} E$), significant wave height ($\Delta H_{s}$); 95th percentile significant wave height ($\Delta H_{s}^{95}$); number of significant wave height events above the 95th percentile with 48 h separation ($\Delta N_{{H_{s}^{95} }}$); mean wave period ($\Delta T_{m}$); mean wave direction ($\Delta \theta_{m}$); storm surge magnitude ($\Delta \eta^{95}$) and number of storm surge events ($\Delta N_{{\eta^{95} }}$). All quantities are shown at coastal locations and presented as bar charts of positive and negative changes.

As noted above, a number of physical processes will contribute to the observed trends in the recession/progradation,$\Delta C_{D}$ data. At short time scales, individual storm occurrence will result in episodic erosion/accretion of sandy beaches. As trends are taken over multiple decades these episodic events will likely not be captured by the present datasets. At medium time scales (2–10 years), changes in storminess such as those associated with climate indices (e.g. El Niño)^24^ will generally also be averaged out in the trend analysis. Our focus here is on the long term recession/progradation, which will primarily be determined by sea level rise and the sediment budget (including fluvial inputs) in each coastal compartment^5,52,53^. However, long term changes in oceanographic parameters, such as waves and storm surge, can also be an important contributor to shoreline recession/progradation^53,54^. For instance, longshore sediment transport is a function of mean wave conditions. Hence, long term changes in mean wave conditions will have an impact on the sediment budget in a coastal cell^5,27^. Similarly, more frequent and more severe storms could result in post-storm beach recovery (a slow process) not being able to keep up with repeated, frequent storm erosion (a rapid process), in itself leading to shoreline recession in the long term^54,55^. It is these secondary impacts on recession/progradation that we are investigating here by trying to identify linkages to trends in the wave and storm surge conditions.

Fluvial processes at the mouths of rivers and human intervention are likely to be much larger than any change due to non-stationary wave or storm surge processes^56–59^ and such processes are clear in the data (Fig. 3 and Supplementary Figs. S8–S11). Therefore, in an effort to remove these influences, in Figs. 4, 5 and Supplementary Figs. S12–S20, only values within the range $\pm 5$ m/year are shown. Values of larger magnitude were excluded from the graphs and analysis. A range of other possible limits ($\pm 2$,$\pm 3$,$\pm 10$ m/year) were also tested, yielding similar results. We have chosen North and South America as an illustrative example to include in the main paper (Figs. 4 and 5) (other coastlines in the Supplementary Material). South America, in particular, is a convenient illustration as it has long and relatively linear east and west coasts. These coasts also have quite different trends in wave properties, thus allowing a qualitative assessment of the impact of changing wave properties on sandy shorelines.

As noted previously the west (Fig. 4) and east (Fig. 5) coasts of South America are characterized by “bursts” of recession/progradation (Figs. 4a, 5a). As is clear in Fig. 1, Supplementary Figs. S1 and S3, and previously reported in a number of studies^16,49,60^, the Southern Ocean is a region which has experienced an increase in wave energy over recent decades. This is evident in Fig. 4b–d as the increase in coastal wave energy flux and significant wave height along most of the western coastline of South America. This is the result of swell propagating from the generation regions in the Southern Ocean. The magnitude of the trend in wave energy flux and significant wave height ($\Delta C_{g} E$,$\Delta H_{s}$ and $\Delta H_{s}^{95}$) decreases towards the north (latitudes north of 0^0^ show almost no trend) (Fig. 4b–d). This is also evident in the mean wave period ($\Delta T_{m}$) (Fig. 4f), which shows an increase in wave period south of − 25° and no consistent sign north of this area. This is associated with the penetration of Southern Ocean swell into the Pacific basin and changes in the local wave climate of the trade wind belts^61^. The wave climate along the coast is also characterized by a small counter clockwise rotation of the mean wave direction ($\Delta \theta_{m}$) (Fig. 4g) south of approximately 0°. Again, this is associated with the increases in Southern Ocean swell and the southward movement of Southern Ocean low pressure systems in recent decades^62^.

In contrast to the consistent changes in wave conditions along the coast, storm surge changes are less uniform (Fig. 4h). There appear to be increases in the extreme south, although this region of Patagonia has a very complex coastline which may not be well resolved by the storm surge model. Further north in the equatorial regions there is a consistent increase in storm surge values ($\Delta \eta^{95}$).

There is not an obvious relationship between the “bursts” of recession/progradation evident in the coastal shoreline dataset and the changes in wave or storm surge conditions. Whereas the wave parameters (Fig. 4b–d) show increases moving south, the shoreline changes show the opposite behaviour. South of approximately − 20°, the coastline appears less dynamic with smaller values of recession/progradation (Fig. 4a). The fact that there is not a clear spatial relationship between the changes in waves and storm surge and recession/progradation indicates that, for these coastlines, trends in waves and storm surge properties are not (yet) significant.

The eastern coast of South America (Fig. 5) shows similar behavior for recession/progradation to that of the west coast. There are “bursts” of recession/progradation along most of the coastline (Fig. 5a), as already seen on the western coast. Table 1 indicates that 42% of sandy beaches on the west coast are receding compared to 49% on the east coast. Although there are a greater percentage of beaches receding on the east coast, the wave conditions show much smaller increases in the magnitude of wave properties compared to the west coast (Fig. 5b–d). This is because, this coastline is less exposed to southwesterly swell propagation from the Southern Ocean. There does, however, appear to be stronger trends in storm surge ($\Delta \eta^{95}$) (Fig. 5h) along the eastern coast (compared to the west) with general increases in the magnitude of storm surge north of approximately − 25°. As for the west coast, here too there is no clear relationship between regions where recession/progradation has occurred and changes in wave and/or storm surge conditions.

A clear example of the lack of a relationship between changes in the magnitudes of wave and surge properties and shoreline recession/progradation is shown in Fig. 5 for the east coast of North America. This coastline shows almost no change in wave climate south of approximately + 45° but there are changes in the storm surge. In contrast to South America (and the other coastlines), however, the recession/progradation are highly distributed along this full coastline, indicating a very dynamic coastline. This occurs despite the fact that there has been no significant change in wave climate and the occurrence of both positive and negative surge trends along extended stretches of this coastline. The west coast of Africa (Supplementary Fig. S16a), similarly shows a dynamic coastline with high values of recession/progradation distributed along the full coastline. In contrast, the wave conditions show almost no trend north of − 15° and consistent positive trends south of this location.

Table 1 shows the percentages of locations on the western and eastern coastlines of: South America, North America, Africa and Australia and Western Europe, with positive trends of wave and surge parameters, and percentage of sandy beaches receding. As already noted in the bar graphs in Figs. 4 and 5 and Supplementary Figs. S12–S20, all coasts show a similar percentage of receding and progradating sandy beaches. In contrast, most coastlines show that there has been an increase in the magnitudes of wave properties and, to a lesser extent, the magnitudes of storm surge. Australia is an interesting example with the west coast showing 46% of coasts receding and east coast 47%. However, the west coast shows only 35% of locations with an increase in the wave energy flux ($\Delta C_{g} E$), whilst the east coast shows 99% of locations with an increase in wave energy flux. The surprisingly low percentage of locations with an increase in wave energy flux on the west coast can be explained by examination of Fig. 1. The northern half of this coastline is oriented north-east/south-west. This means it is somewhat protected in terms of Southern Ocean swell. As it is the Southern Ocean where changes in wave conditions are most significant, this coast appears to respond more to local wave events than Southern Ocean swell. Despite the very different changes in wave climates on the east and west coasts of Australia the recession/progradation data are not appreciably different.

## Summary and conclusions

In recent decades, a number of studies have shown globally increasing values of ocean wave height and period. The present paper also demonstrates that, coastal storm surge has shown regionally consistent changes, with extended stretches of coastlines showing increases or decreases. As sandy coastlines are dynamic systems, with recession/progradation responding to waves and surges, it is often speculated that these historical changes and projected future changes of wave and surge conditions, may be important drivers of shoreline recession/progradation. In this study, we have attempted to determine if there are measurable global scale linkages between changes in sandy shoreline recession and progradation and historical changes in waves and storm surge.

As we focus on global-scale changes, we need to use measured and modelled datasets of global shoreline recession/progradation, changes in wave conditions and storm surge. We do not claim that such an analysis will be appropriate to describe the dynamics of an individual beach. Rather we are seeking to investigate if there are large scale changes in recession/progradation which may be linked to similarly large-scale changes in wave and/or storm surge conditions.

We use two independent global datasets of sandy shoreline recession/progradation and show they yield similar results. We also validate these global datasets with an independent, validated, higher-resolution regional shoreline change dataset (see “Limitations and validation” section). We use a global wave dataset and assume that trends in offshore wave conditions will result in similar trends in nearshore waves. To validate this assumption, we compare offshore trends from the global deep-water wave hindcast with a high-resolution regional nearshore wave hindcast (see “Limitations and validation” section). This comparison shows that the assumption does hold adequately for the purposes of our study. The global storm surge model dataset used has previously been validated against an extensive network of global tide gauge stations^37^.

The results are compared for nine continental coastlines. We compare measured changes in sandy shoreline recession/progradation along these coastlines over an approximately 30-year period with corresponding trends in wave energy flux, wave height, mean wave period, wave height extremes and number of such extremes and changes in wave direction, storm surge magnitudes and the number of storm surge events. We find no obvious linkages between regions where there are strong trends in wave and/or surge quantities and the large-scale dynamics of these coastlines. For instance, west coasts of continents in the southern hemisphere show stronger positive trends in wave conditions than east coasts, largely driven by changes in Southern Ocean swell. However, there is no clear evidence that the sandy coastlines along these west coasts are more dynamic than those along the east coasts. This lack of correlation also extends to wave direction (a primary driver of longshore sediment transport), where the changes in deepwater wave direction appear uncorrelated with the shoreline recession/progradation. Note, however, that refraction will mean that offshore and nearshore wave direction may differ substantially.

A good example of of the lack of clear correlation is a comparison of the west coast of South America (strong positive trends in wave conditions) and the east coast of the North America (relatively small trends). Despite this, the sandy coastlines along the east coast of North America actually show larger changes than those along the west coast of South America.

We do not infer that beaches are not, or will not, respond to changes in wave and storm surge conditions. Indeed, recent studies have shown that changes in wave climate, as a result in El Niño do impact medium-term coastal erosion for the Pacific^24^. Rather, we conclude that the available datasets do not show clear forcing/response linkages between long-term shoreline change and changes in waves and storm surge over the past three decades. Changes in significant wave height have been less than 1 cm/year (30 cm over 30 years) and storm surge less than 0.1 cm/year (3 cm over 30 years) over this period. Such changes may be too small to yield a measurable change in sandy shorelines, noting other processes impacting the coastline (e.g. sea level rise, terrestrial sources of sediment, alongshore gradients in longshore sediment transport). It should also be noted that the present sandy shoreline change datasets are based on total observed shoreline change, which would include non-wave/surge driven phenomena, such as fluvial sediment loads, land reclamation and other local sediment sinks/sources. Importantly, shoreline position is significantly determined by the sediment budget available in an area. It is not yet possible to separate out these additional contributions to shoreline change from the satellite observations. Therefore, it is also possible that these higher level contributions mask any clear relationship between wave/surge forcing/response linkages over the last 30 years.

Morim et al.^27^ projects near coast changes in $H_{s}$ by 2100 of less than 5% (or 0.15 m for a nearshore mean $H_{s}$ of approximately 3 m). As these changes are actually slightly smaller than the historical changes, it is probable that clear changes as a result of changing wave climate will also not be visible, even by 2100. In contrast, sea level rise over this same period is projected to impact sandy coastlines in a very noticeable manner^3^.

## Methods

### Data

The analysis uses several global and regional datasets. These datasets are described below and their limitations are outlined in the Limitations and Validation section.

#### Liu et al. global wave hindcast

The Liu et al.^33^ dataset is a wave hindcast generated with the third-generation spectral wave model, Wavewatch III^63^. The model was forced with ERA5 winds^34^ and uses the observation-based source term parameterization ST6^40^. The grid resolution of the dataset is 0.25° × 0.25°. Output data from the hindcast were available at 3 h intervals. Although the model does include finite depth processes such as refraction, shoaling and bottom friction, the resolution is such that this is effectively a deep-water wave dataset. In order to apply these gridded data at coastal locations, the closest Liu et al. global grid point to the shoreline was assigned as representative of shoreline conditions.

#### Liu et al. regional wave hindcast

The Liu et al.^40^ regional hindcast provides historical wave climate data for Bass Strait and South-East Australia (domain bounded by latitudes − 35° to − 45° and longitudes 137° to 155°). The hindcast uses the Wavewatch III model with the same physics as the global hindcast of Liu et al.^33^, within which this regional model is nested. The regional model uses an unstructured grid for Bass Strait and South-East Australia, with a spatial resolution as fine as 250 m in coastal regions. The model has been extensively validated against a network of 13 coastal buoys^40^, with data available for the time period 1981–2020. For the present application, these data have been used to verify that trends in deep-water wave quantities obtained from the global model^33^ are generally consistent with the trends in the hindcast data at near-coast locations (see “Limitations and validation” section and Supplementary Fig. S21). In order to apply these gridded data at coastal locations, the closest Liu et al. regional grid point to the shoreline was assigned as representative of shoreline conditions..

#### GTSR global tide and surge reanalysis

The GTSR dataset^36^ was generated with the Global Tide and Surge Model (GTSM), an implementation of Delft3D-FM^64^. The model uses wind and atmospheric pressure fields generated by the ERA-Interim global atmospheric reanalysis^48^. For validation, GTSR data have been compared with observed sea levels from 472 global tide gauges^36,37^. The dataset is available with temporal resolution of 10 min and uses a series of grids with resolution from 50 km in the deep ocean to 5 km in coastal areas^36,37^. For the present application, time series at the 9866 DIVA coastal locations were used^39^.

#### Updated Delft ($\Delta C_{D}$) dataset

The *Delft*^2^ dataset is a global analysis of the state of world beaches using available optical satellite images captured since 1984. The dataset presents a global-scale evaluation of the dynamics of sandy shorelines and estimated shoreline change rates employing 33 years of Landsat image archives (1984–2016). Shoreline change rates in units of m/year are available for transects with an alongshore spacing of 500 m. The dataset contains information of shoreline change rates at more than 1.7 million points along world coastlines and classified as either sandy or non-sandy. In this study we used an updated version of this dataset that excludes some contentious sandy locations which were present in the original data set.

#### Mentaschi et al. ($\Delta C_{JRC}$) dataset

The *JRC*^38^ dataset is also a global satellite observation-based analysis of coastal morphodynamics from 1984 to 2015 (32 years). The availability of satellite data is not uniform for all the locations, and so the time horizon of the analysis is not the full 32 years at all locations. The dataset is based on the analysis of the high-resolution Global Surface Water Explorer (GSWE)^65^ database. It provides information about land–water transitions using dry–wet and wet–dry definitions. The estimation of land losses and gains were obtained from the changes in the presence of water along more than 2 million virtual transects. The spatial resolution of this dataset is 30 m in the cross-shore direction and 250 m in long-shore direction. Unlike the *Delft*^2^ dataset, *JRC*^38^ does not provide information on beach type and has been used in the present study as a validation dataset.

#### Geoscience Australia ($\Delta C_{GA}$) dataset

Bishop-Taylor et al.^51^ produced a regional dataset around the Australian coastline containing annual change rate values of recession/progradation from 1988 to 2019. The dataset utilizes a combination of satellite data and tidal modelling. This dataset has very fine-scale along-coast resolution of 30 m. As the dataset has been extensively validated against in situ measurements, (a total of 330 validation transects, each with greater than 10 years of coastal monitoring data) it has been used in the present context to validate the global shoreline change datasets^2,38^ (see “Limitations and validation” section below and Supplementary Fig. S20).

### Trend calculation data periods

Statistical trend analysis techniques were applied to investigate possible changes in mean and percentile values of the waves and storm surge data at coastal locations. For these purposes, yearly mean and higher percentile values have been used. Both linear regression and the nonparametric Theil–Sen estimator^8,13,42,66^ were used. The Theil–Sen estimator is often regarded as a more robust approach with less sensitivity to the impact of potential outliers. It calculates the median of slopes for each distinct pair of yearly mean and percentile values^49^. In order to compare the various datasets, a common time period for the trend analysis was required. The recorded time periods for the available datasets are as follows:

- Liu et al.^33^ global wave hindcast—1979 to 2018
- Liu et al.^40^ regional wave hindcast—1981 to 2020
- GTSR^36^ global surge reanalysis—1979 to 2014
- *Delft*^2^ global shoreline change dataset—1984 to 2016
- *JRC*^38^ global shoreline change dataset—1984 to 2015
- *Geoscience Australia*^51^ regional shoreline change dataset—1988 to 2019.

As a result, a common time period of 1984 to 2016 was chosen for the analysis. This means that the GTSR (1984–2014) $\Delta \eta^{95}$ and $\Delta N_{{\eta^{95} }}$ and *Geoscience Australia* (1988–2016) $\Delta C_{GA}$ change rates were calculated over slightly shorter periods.

### Wave and surge trend calculations

The time series of wave parameters at assigned coastal locations for the period 1984–2016 were extracted from the global Liu et al.^33^ and regional Liu et al.^40^ datasets. To investigate the changes in wave conditions, annual time series of mean and higher percentile (95th, 98th, 99th, 99.5th) time series were calculated for each coastal location. Trend analysis using both linear regression and Theil–Sen estimates^42,66^ were applied to these data, generating the quantities: $\Delta C_{g} E$,$\Delta H_{s}$,$\Delta H_{s}^{95}$,$\Delta T_{m}$ and $\Delta \theta_{m}$. In addition, extreme events were determined as occasions on which the time series exceeded the 95th percentile but with such events separated by at least 48 h. The number of these events in each year were determined and trend analysis conducted to determine trends in these extreme events, $\Delta N_{{H_{s}^{95} }}$. For the GTSR^36^ data, surge events were similarly identified as cases where the water level exceeded the 95th percentile, separated by at least 48 h. Trend analysis was then applied to determine changes in magnitude ($\Delta \eta^{95}$) and number ($\Delta N_{{\eta^{95} }}$) of these events. Note that the linear regression and Theil–Sen estimates^42,66^ of trend were very similar for all quantities. Hence, all results presented have been calculated with linear regression.

### Shoreline change (recession/progradation) rates

The *JRC*^38^ shoreline change dataset does not directly define a coastline location or change in coastline. Rather, it provides information about the land–water transition point using dry–wet and wet–dry definitions^2^ in six categories, with three of these related to costal recession: permanent land to permanent sea, $Y_{ls}$, permanent land to active zone, $Y_{la}$, and active zone to sea, $Y_{as}$. Also, progradation categories include permanent sea to permanent land, $Y_{sl}$, permanent sea to active zone, $Y_{sa}$, and active zone to land, $Y_{al}$. Therefore, the total net value of the changes in shoreline position over the recording period at each location, can be estimated as:

$${\text{Net}}\,{\text{shoreline}}\,{\text{change}} = \left( {Y_{sl} + Y_{sa} + Y_{al} } \right) - \left( {Y_{ls} + Y_{as} + Y_{la} } \right).$$

The shoreline change rate $\Delta C_{JRC}$ with units (m/year) can then be determined by dividing these values by the recording period for each coastal location.

Both the updated *Delft*^2^ and *Geoscience Australia*^51^ datasets directly provide values of shoreline change rate (m/year) $\Delta C_{D}$ and $\Delta C_{GA}$, respectively. In both cases, the change rates were determined from linear region of annual values of shoreline position.

Since the focus of this study is on sandy beaches, and as the *JRC*^38^ dataset does not provide a classification of beach type, we have used the coordinates of sandy beach locations in the updated *Delft*^2^ dataset as a reference. The closest locations in the *JRC* dataset to these coordinates were taken as sandy beaches, provided the distance between the locations was less than 1 km. A similar approach was also applied to classify the beach-type for the finer scale regional *Geoscience Australia*^51^ dataset. Hence, in the figures which show comparisons between the various shoreline change datasets (Fig. 3, Supplementary Figs. S8–S11, S21) if there is no *JRC* or *Geoscience Australia* data point within 1 km of the *Delft* sandy location, both the *Delft* sandy location and any non-assigned *JRC* or *Geoscience Australia* data location are removed from this comparison. In this way, the comparisons have the same number of datapoints, facilitating a meaningful comparison. As a one-to-comparison of this type is not possible for the plots that show shoreline change rates compared to wave and storm surge parameters (Figs. 4, 5, Supplementary Figs. S12–S20), for these comparisons, all *Delft* sandy locations are retained. Therefore, there will be some differences between the recession/progradation rates shown for the *Delft* data in these two different forms of comparisons. For the plots which show comparisons between shoreline change rates and wave/surge trends (Figs. 4, 5, Supplementary Figs. S12–S20), the shoreline recession/progradation data $\Delta C_{D}$ is filtered to retain only values $\Delta C_{D} < \pm 5$ m/year. This is done to remove large values of shoreline change which are assumed associated with fluvial processes or human impacts (beach nourishment etc.).

## Limitations and validation

The global-scale nature of the present analysis means a number of assumptions/simplifications are necessary to make the problem tractable. These are summarised below.

### Nearshore wave trends

A high resolution global coastal wave hindcast is not available and it is doubtful that it is computationally feasible to develop such a resource in the foreseeable future. As an alternative, we have used the deep-water data of Liu et al.^33^. This dataset is on an 0.25° regular global grid. The closest deep-water data point to the shoreline was assigned as representative of shoreline conditions. Our assumption is that, although the magnitude of wave properties (e.g. $H_{s}$,$T_{m}$, $\theta_{m}$) will change in transformation from these offshore locations to the coast, the distribution along the coast of deep-water trends will be representative of nearshore trends. That is, for example, a positive deep-water trend in $H_{s}$ will generally result in a positive nearshore trend. Although there will be specific local locations where the nearshore bathymetry and coastline will result in significant differences, at global scale we believe this is a reasonable assumption.

To test this assumption, we have compared the trends in the deep-water Liu et al.^33^ dataset with the data from a high-resolution regional model^40^. Both models use the same wind forcing and physics and the regional model is nested within the global model. Therefore, the only differences between the models are the spatial and temporal resolution and the finite depth propagation, transformation, bottom friction and depth-limited breaking in the coastal regions of the regional model. The domain of the high-resolution regional model is Bass Strait and south-east Australia (see Supplementary Fig. S21). This regional model has been extensively validated in deep water against altimeter data^67^ and in nearshore regions against a network of 13 coastal wave buoys^40^. This is a particularly demanding test of our assumptions, as the area has complex geomorphology. In Supplementary Fig. S21, the wave conditions along the coastline of continental Australia from west to east are examined. The west is exposed to swell from the Southern Ocean. In the central regions, the coast is protected by the island of Tasmania to the south and Bass Strait between the continent of Australia and Tasmania is an extensive area of relatively shallow sea (less than 150 m). To the east, the coast is in the lee of Tasmania and then further east is exposed to local winds and swell form the southern Pacific.

Supplementary Figure S21d,e show the trends in wave energy flux for the regional ($\Delta C_{g} E(r)$) and global ($\Delta C_{g} E(g)$) models, respectively. The regional ($\Delta H_{s} (r)$) and global ($\Delta H_{s} (g)$) trends in significant wave height are also shown in Supplementary Fig. S21f,g, respectively. As expected, there are differences between the regional and global models and the much finer along coast resolution in the regional model provides greater fine-scale variability. Despite these differences, the magnitudes of the trends are comparable and the general along-coast structure of the trend is similar. In the western regions (135° to 145°) both models show positive trends for $C_{g} E$ and $H_{s}$. Within Bass Strait (144° to 146°) the magnitudes of the trends decrease due to reduced penetration of Southern Ocean swell. In the lee of Tasmania (146° to 150°) both models show the trends in $C_{g} E$ reducing to approximately zero and that in $H_{s}$ becoming negative. Along the east coast of Australia (> 150°) both models show strong positive trends as the wave climate becomes more exposed to oceanic swell from the Pacific and Southern Oceans.

These comparisons provide a degree of confidence that application of the global wave datasets to coastal regions does not invalidate the conclusions regarding trends in nearshore wave conditions over the past decades.

It should be noted that, although the regional model of Liu et al.^40^ is a state of the art finite depth spectral wave model, which has been extensively validated, its validity in very shallow water has not been explored.

### Shoreline change dataset

The *Delft*^2^ shoreline change (recession/progradation) dataset has been used in this study. Obtaining such data from Landsat satellite images on a global scale is a demanding task, with the challenges of automatic detection of the land/water boundary, removal of tide and swash effects and the need to determine sub-pixel changes, all significant issues. In order to validate these results, we have compared results with an alternative global dataset^38^. Comparisons for a range of global coastlines appear in Fig. 3 and Supplementary Figs. S8–S11. Although there are differences, presumably due to different processing techniques, datasets and resolution, the same general trends are evident in both datasets.

To further validate the *Delft*^2^ data we have compared with the higher resolution *Geoscience Australia*^51^ dataset for the same region of south-east Australia considered above and shown in Supplementary Fig. S21. Importantly, the *Geoscience Australia*^51^ dataset has been extensively validated against in situ data. Supplementary Figure S21a shows the recession/progradation results from the global dataset ($\Delta C_{D}$) and Supplementary Fig. S21b for the regional dataset ($\Delta C_{GA}$). As noted above, both datasets have been filtered to retain only values smaller in magnitude than $\pm$ 5 m/year. The two datasets are quite similar. In particular, both datasets show a dynamic shoreline between latitudes of 144° to 148°, with less activity both east and west of this region.

### Choice of global wave database

The deep water Liu et al.^33^ database is termed a wave hindcast, as it is forced with historical winds. An alternative is to use a global wave reanalysis dataset, which contains similar wind forcing, but also assimilates altimeter wave height data. A widely used reanalysis of this type is ERA5^34^. Supplementary Figure S2 shows values of coastline trends of annual mean values of $H_{s}$ from ERA5. The Liu et al. (hindcast) and ERA5 (reanalysis) can be compared in Supplementary Figs. S1 and S2, respectively. It is clear in these figures that ERA5 has stronger positive trends globally than the Liu et al. data. This is consistent with previous global studies of trends in $H_{s}$^39–41^. As shown by Casas-Prat et al.^68^ these differences occur because of the non-homogeneity of the reanalysis data caused by the assimilation of altimeter data since 1992, making ERA5 data unsuitable for the determination of trends in $H_{s}$. As values of trend are critically important for the present study, the Liu et al. hindcast dataset was adopted.

### Shoreline data visualization

As is clear in Figs. 3, 4 and 5, a characteristic of the shoreline recession/progradation data is the high spatial variability of the data, including changes in sign over relatively short distances (one beach recessing and next prograding). This characteristic means that the data cannot be represented meaningfully, at global scale, in plots such as Figs. 1 and 2. After much testing, the latitudinal bar charts of Figs. 3, 4 and 5 appear to provide the most reasonable representation of such data.

It would be extremely difficult to show the Mediterranean in such a format. Similarly we do not show the southern coast of Australia, other than for the regional validation, Supplementary Fig. S21 (where we show longitudinal bar charts). In terms of the west coast of Africa, we excluded the coast north of the Equator, as this coastline has significant curvature.

### Tropical cyclone impacts

The relatively low spatial resolution of the global wave and storm surge models used in this study means that the impacts of tropical cyclones will not be adequately captured in this analysis. Therefore, results in the tropics and sub-tropics should be treated with caution.

## Supplementary Information

Supplementary Figures.

## Data Availability

All data needed to evaluate the conclusions in the paper are present in the paper and/or the Supplementary Materials. Additional data related to this paper may be requested to the corresponding author.
