# Supplementary material for: An assessment of whether long-term global changes in waves and storm surges have impacted global coastlines
Source: Sci Rep. 2023 Jul 17;13:11549. doi: 10.1038/s41598-023-38729-y (PMC10352243; doi:10.1038/s41598-023-38729-y)
Supplement: Supplementary file 1 — Supplementary Figures. [file 41598_2023_38729_MOESM1_ESM.pdf]

Supplementary Material

An assessment of whether long-term global changes in waves and storm surges have impacted global coastlines

Mandana Ghanavati[1], Ian Young[1], Ebru Kirezci[1], Roshanka Ranasinghe[1,2,3,4], Trang Minh Duong[2,3,4], Arjen P. Luijendijk[3,5]

[1] Department of Infrastructure Engineering, University of Melbourne, Melbourne, VIC 3010, Australia.
[2]Department of Water Science and Engineering, IHE-Delft, P.O. Box 3015, 2610 DA Delft, Netherlands.
[3]Resilient Ports and Coasts, Deltares, P.O. Box 177, 2600 MH Delft, Netherlands.
[4]Water Engineering and Management, Faculty of Engineering Technology, University of Twente, P.O. Box 217, 7500 AE Enschede, Netherlands.
[5]Department of Hydraulic Engineering, Faculty of Civil Engineering and Geosciences, Delft University of Technology, Delft, the Netherlands

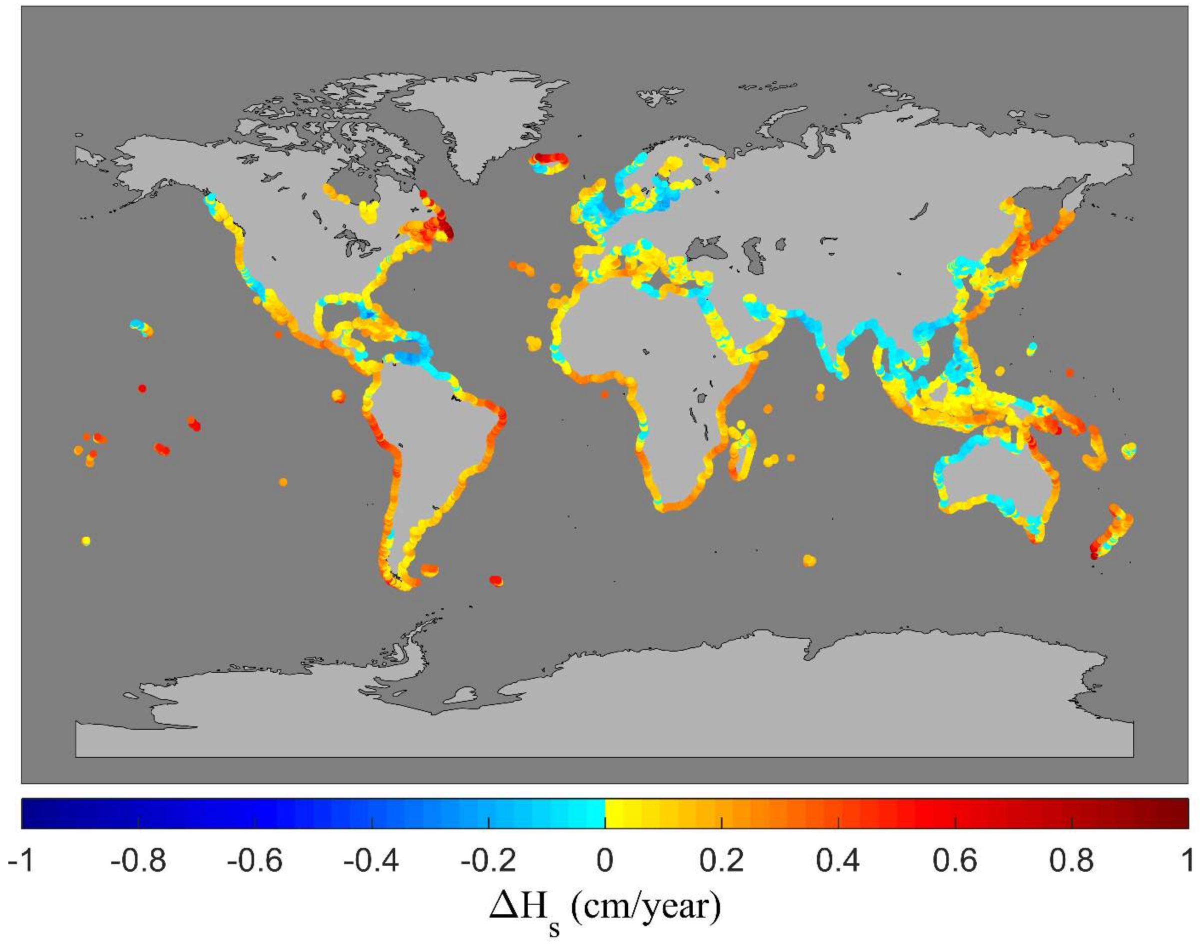

Figure S1. Global distribution of trends in annual mean values of significant wave height (cm/year) at coastal locations for the period of 1984-2016 from the Liu et al. dataset.

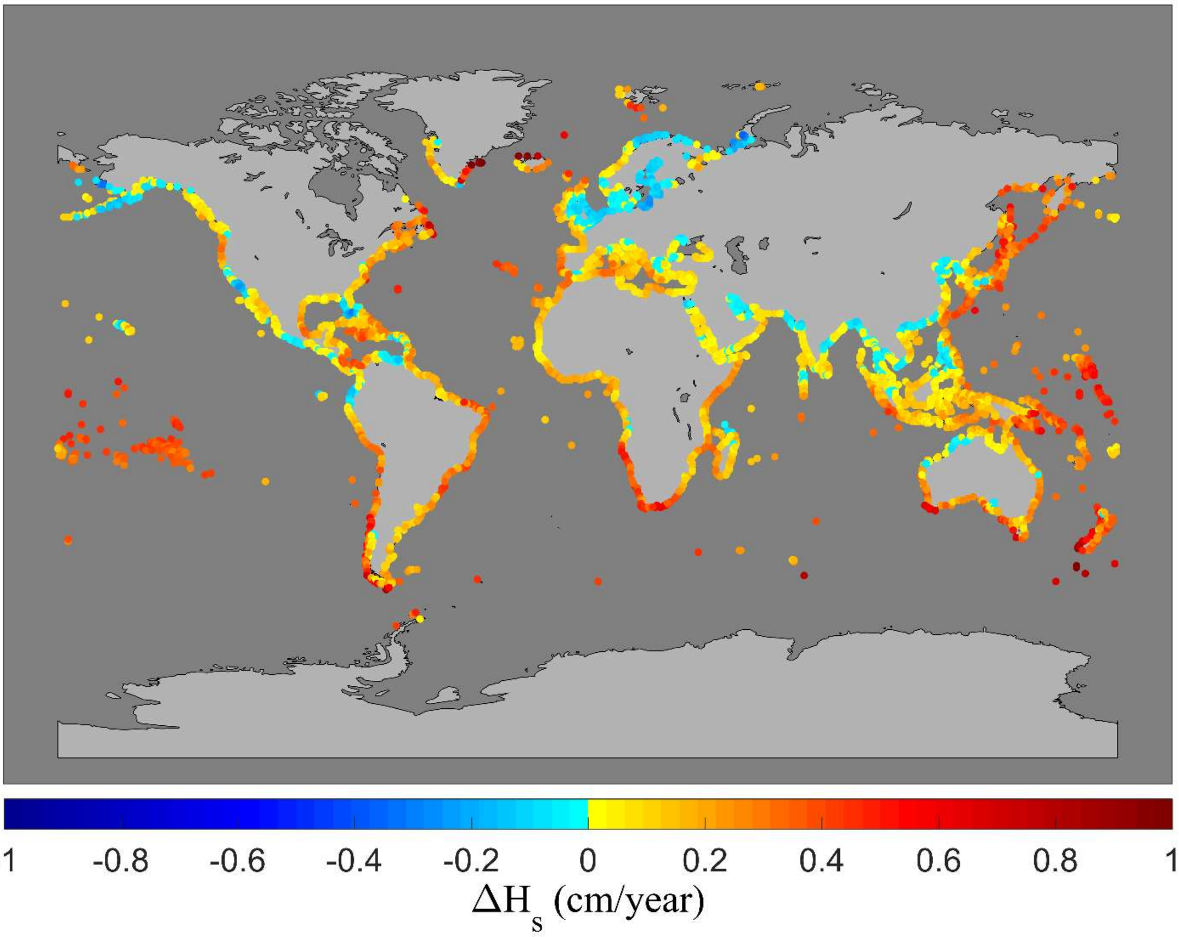

Figure S2. As for Figure S1 but for the ERA5 dataset.

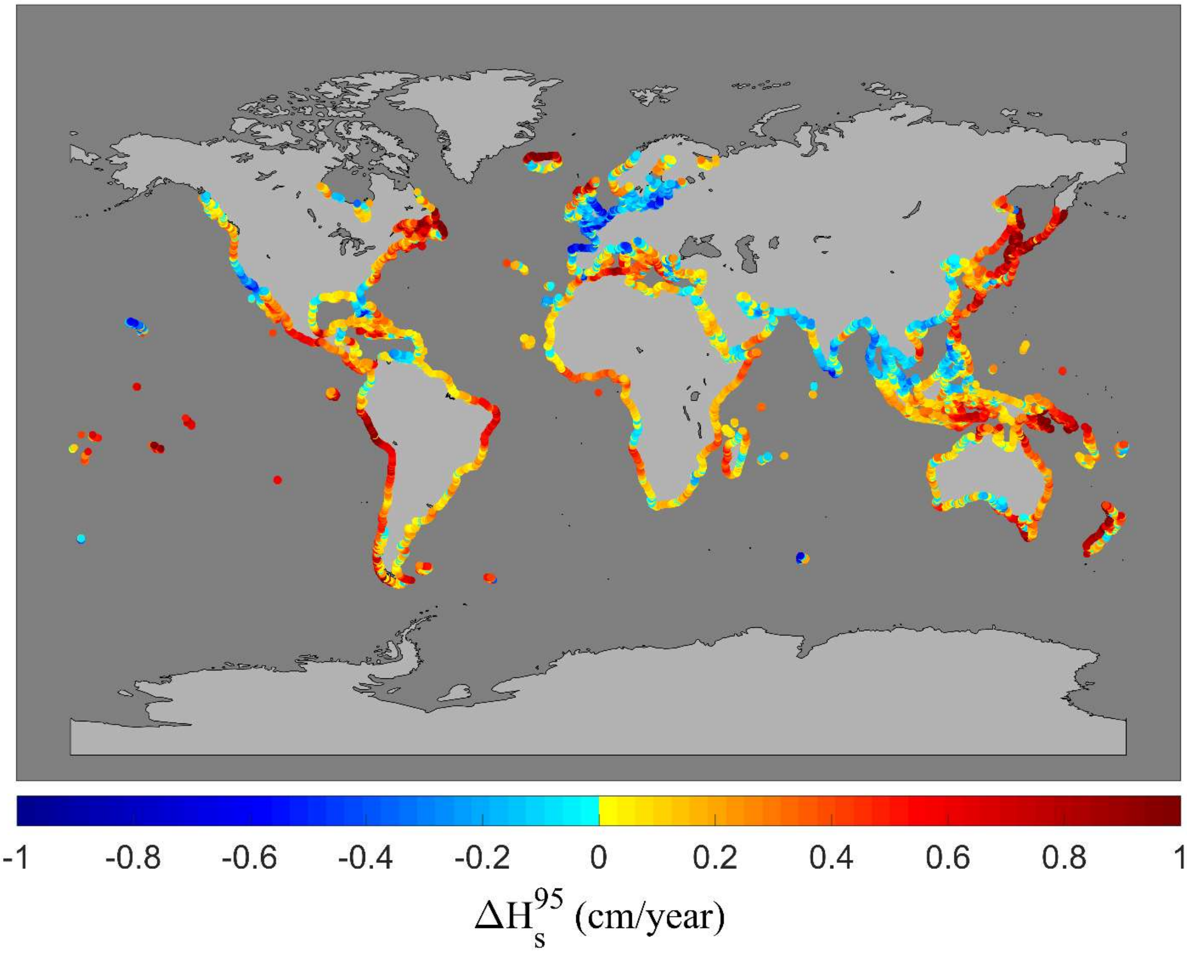

Figure S3. Global distribution of trends in annual mean values of 95th percentile significant wave height ΔH_s^{95} (cm/year) at coastal locations for the period of 1984-2016 from the Liu et al. dataset.

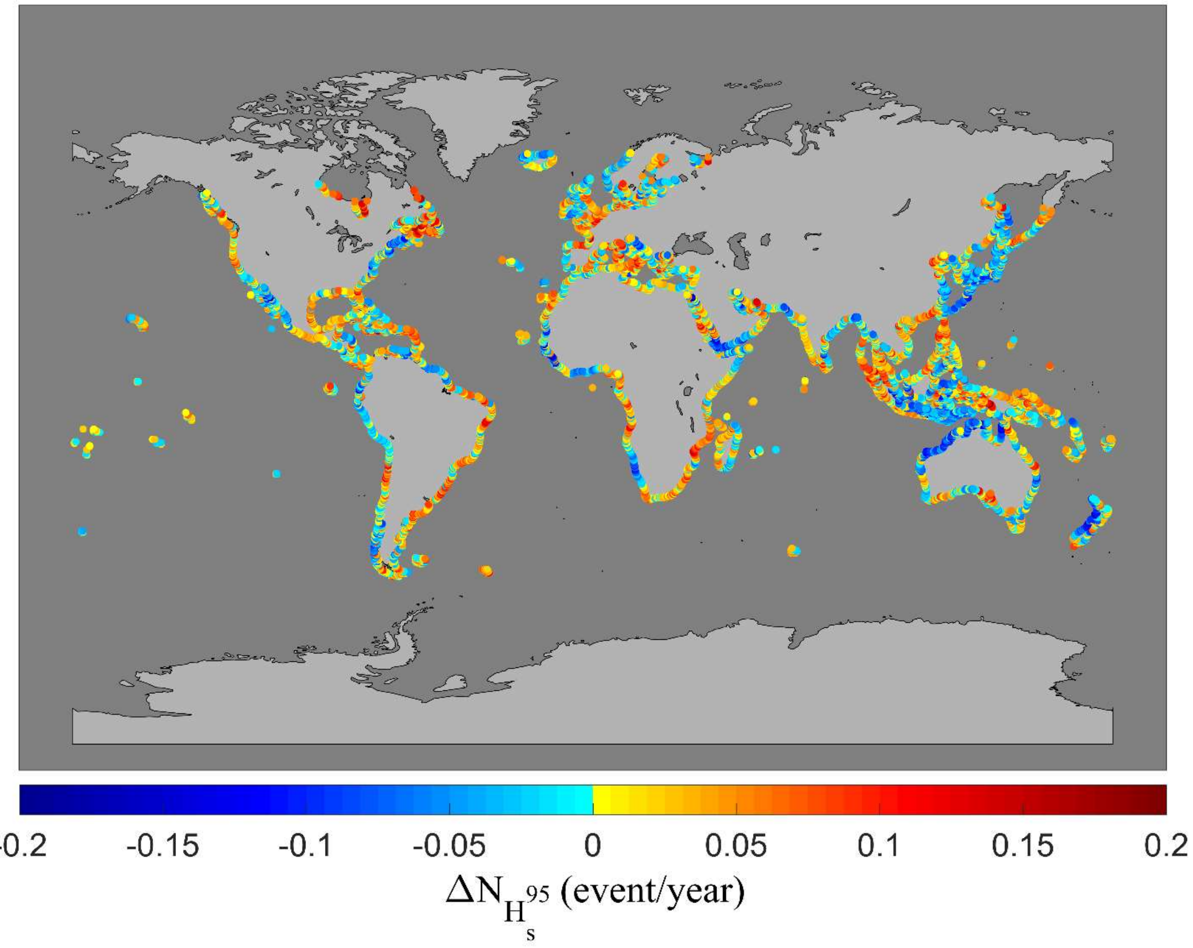

Figure S4. Global distribution of trends in annual values of number of extreme wave height events above the 95th percentile $\Delta N_{H_s^{95}}$ (event/year) at coastal locations for the period of 1984-2016 from the Liu et al. dataset.

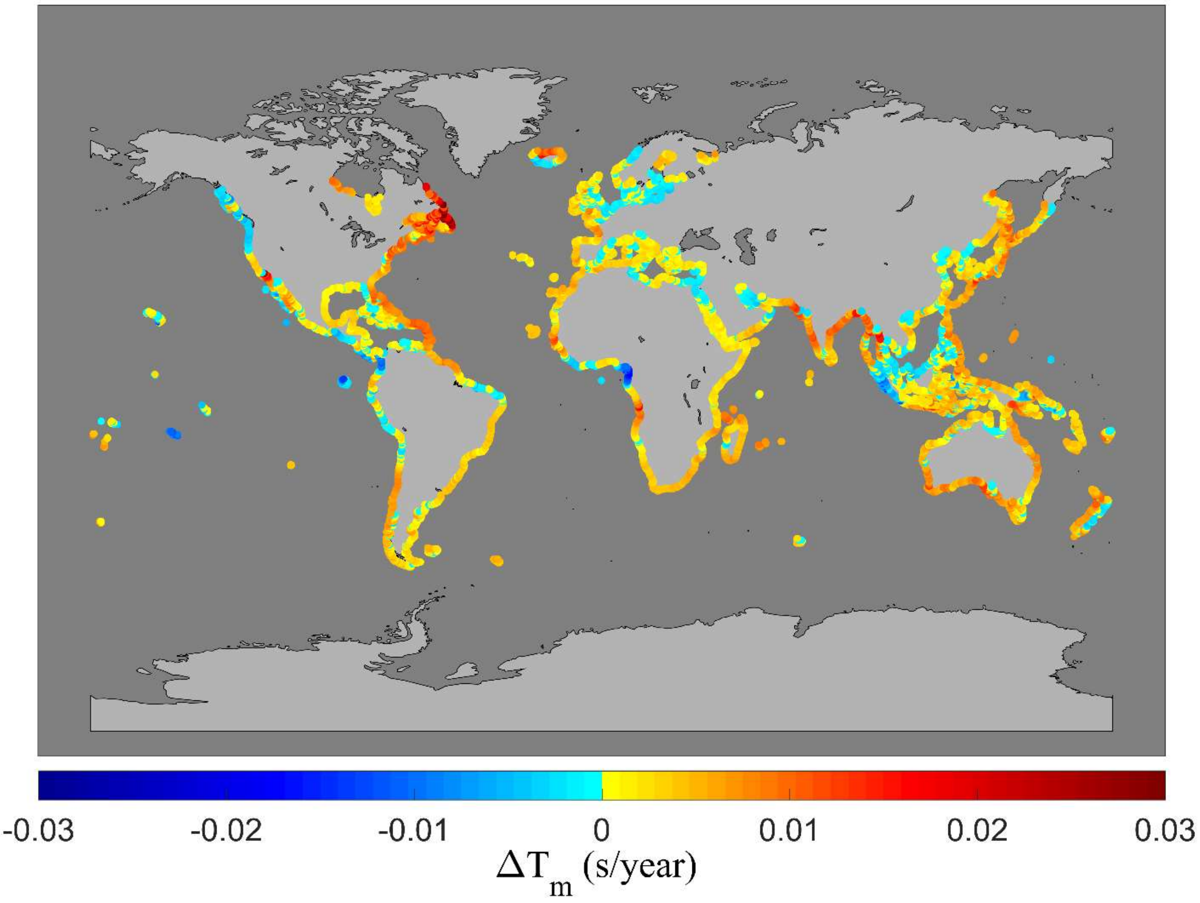

Figure S5. Global distribution of trends in annual mean values of mean wave period ΔT_m (s/year) at coastal locations for the period of 1984-2016 from the Liu et al. dataset.

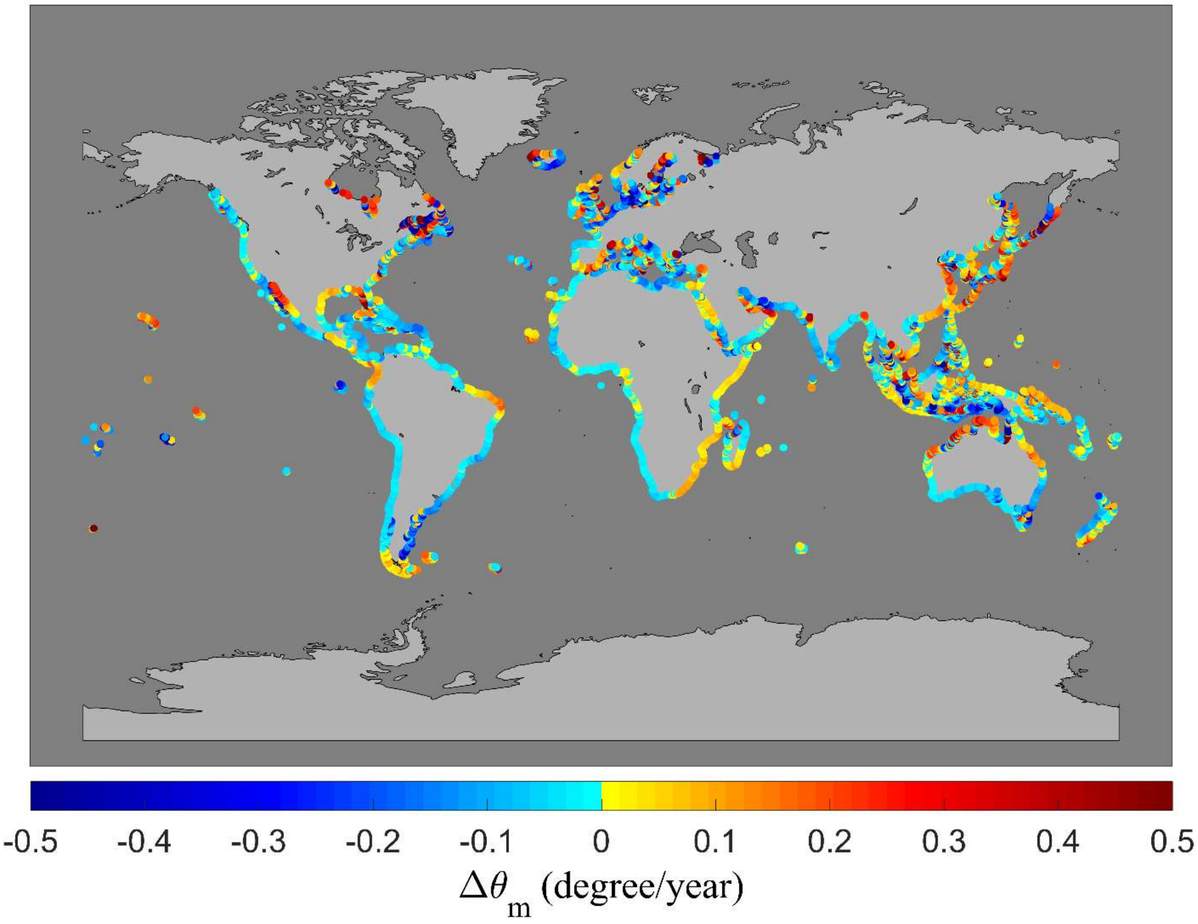

Figure S6. Global distribution of trends in annual mean values of mean wave direction $\Delta\theta_m$ (degree/year) at coastal locations for the period of 1984-2016 from the Liu et al. dataset. Positive (negative) values refer to a clockwise (anti-clockwise) rotation over the period.

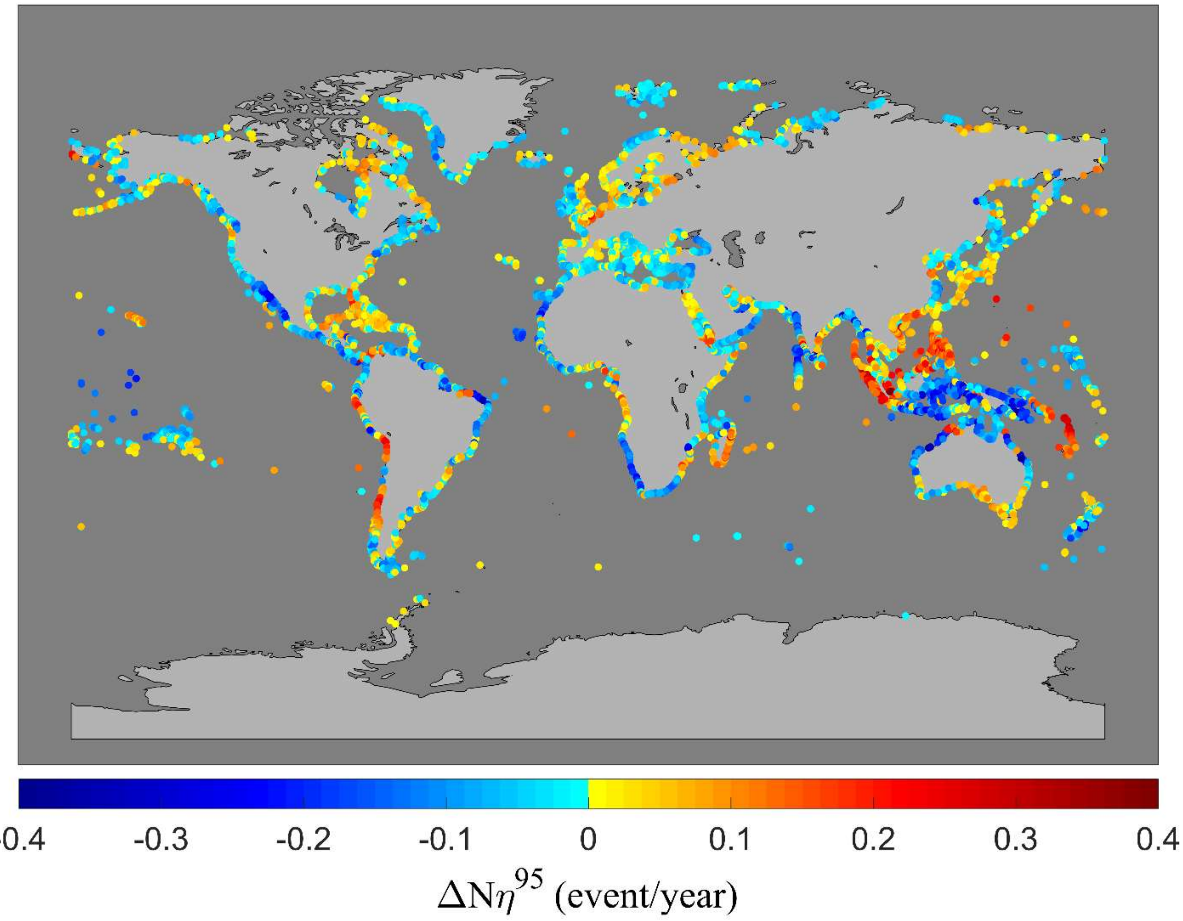

Figure S7. Global distribution of trend in the number of storm surge events, $\Delta N_{\eta 95}$ (95th percentile of water level) (event/year) for the period of 1984-2014 at DIVA locations.

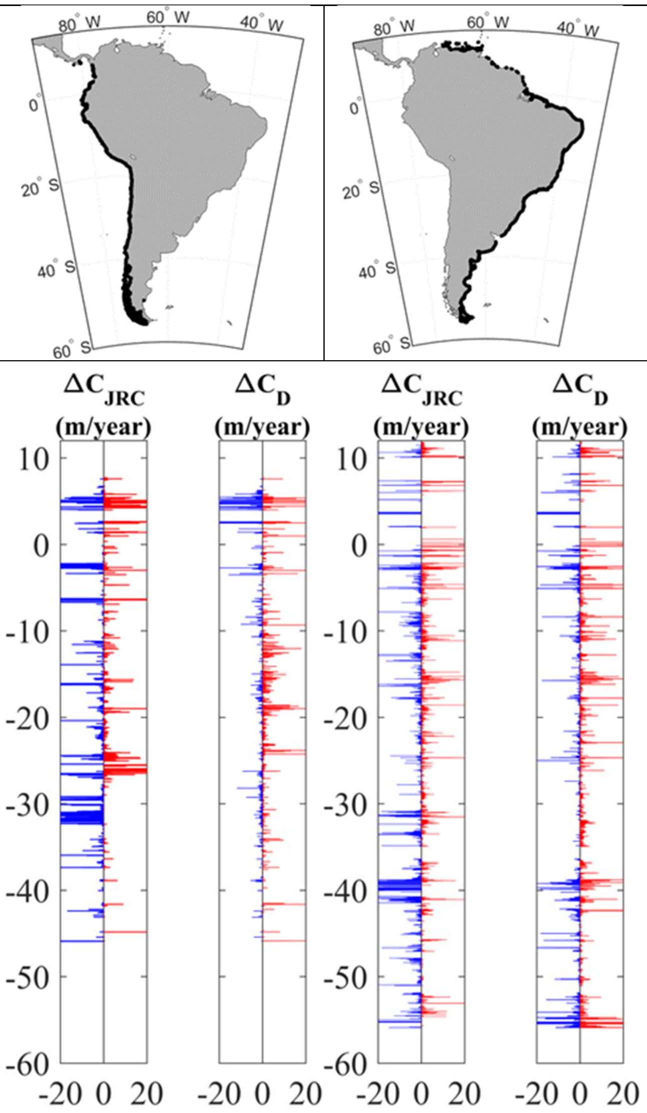

(a) (b) (c) (d)

Figure S8. Latitudinal distribution of shoreline change rate of sandy shorelines for the west coast of South America for (a) *JRC* (ΔC_{JRC}) and (b) *Delft* (ΔC_D) datasets, respectively. Similarly, (c) and (d) show latitudinal distribution of shoreline change rate of sandy shorelines for east coast of South America for (c) *JRC* (ΔC_{JRC}) and (d) *Delft* (ΔC_D) datasets, respectively. The inserts at the top show the continent of South America highlighting the coastline regions considered.

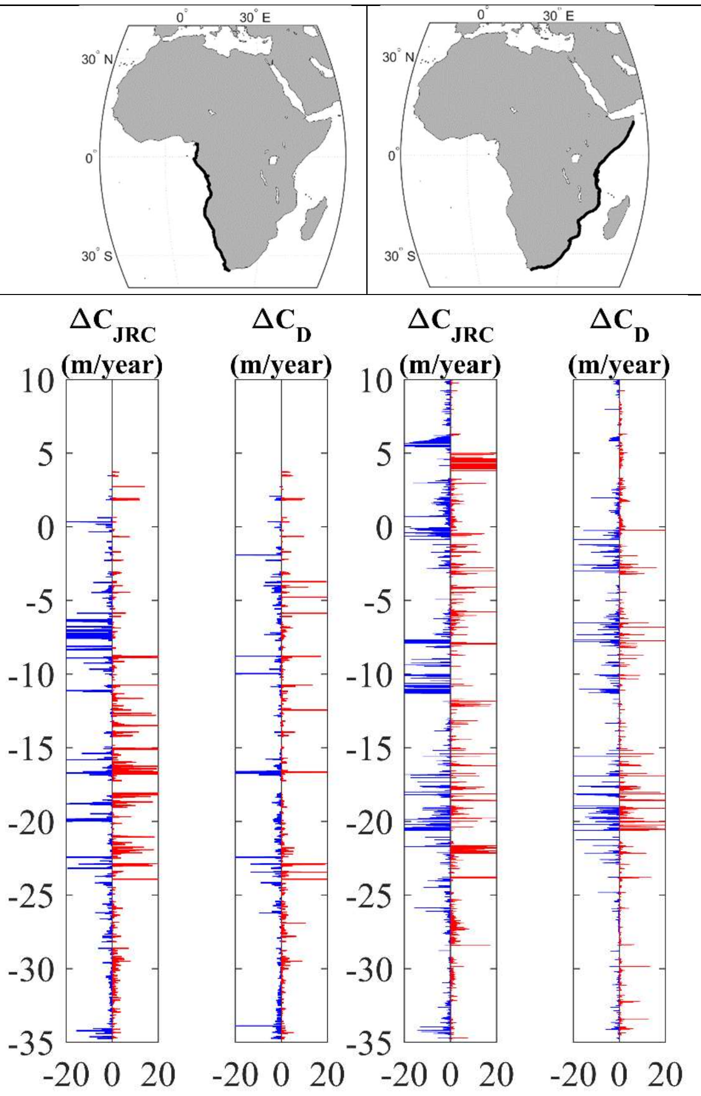

(a)	(b)	(c)	(d)

Figure S9. As for Figure S8 but for Africa.

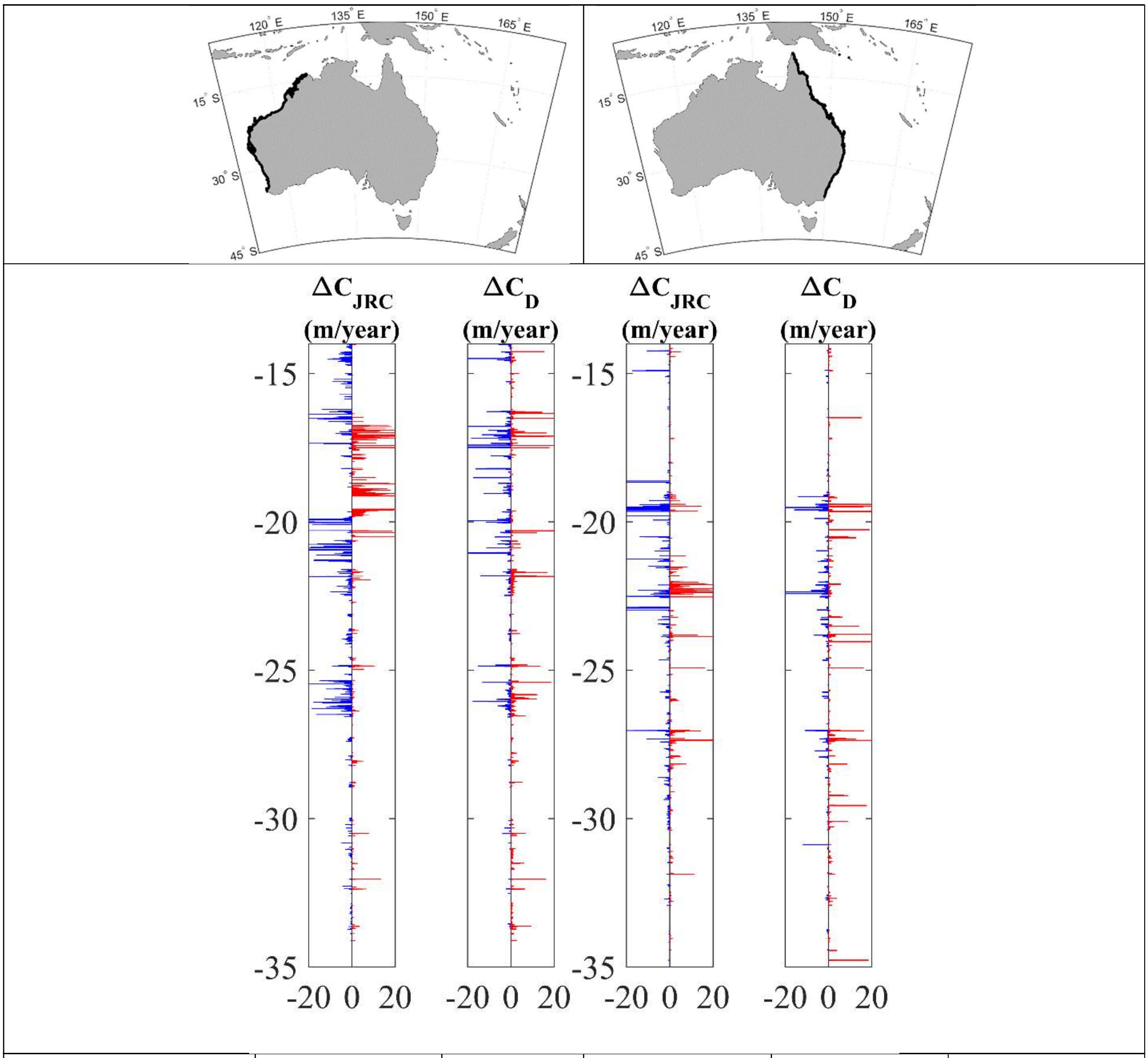

(a) (b) (c) (d)

Figure S10. As for Figure S8 but for Australia.

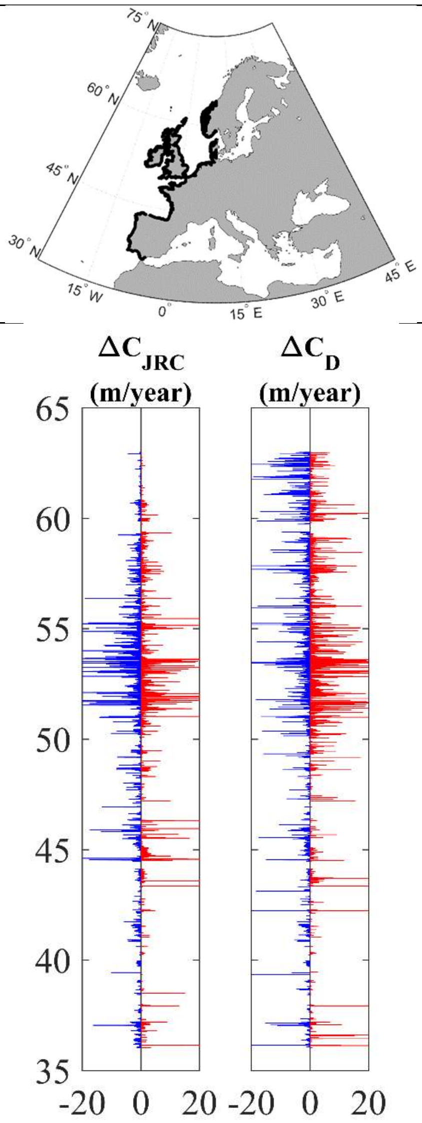

	(a)	(b)	

Figure S11. As for Figure S8 but for western Europe.

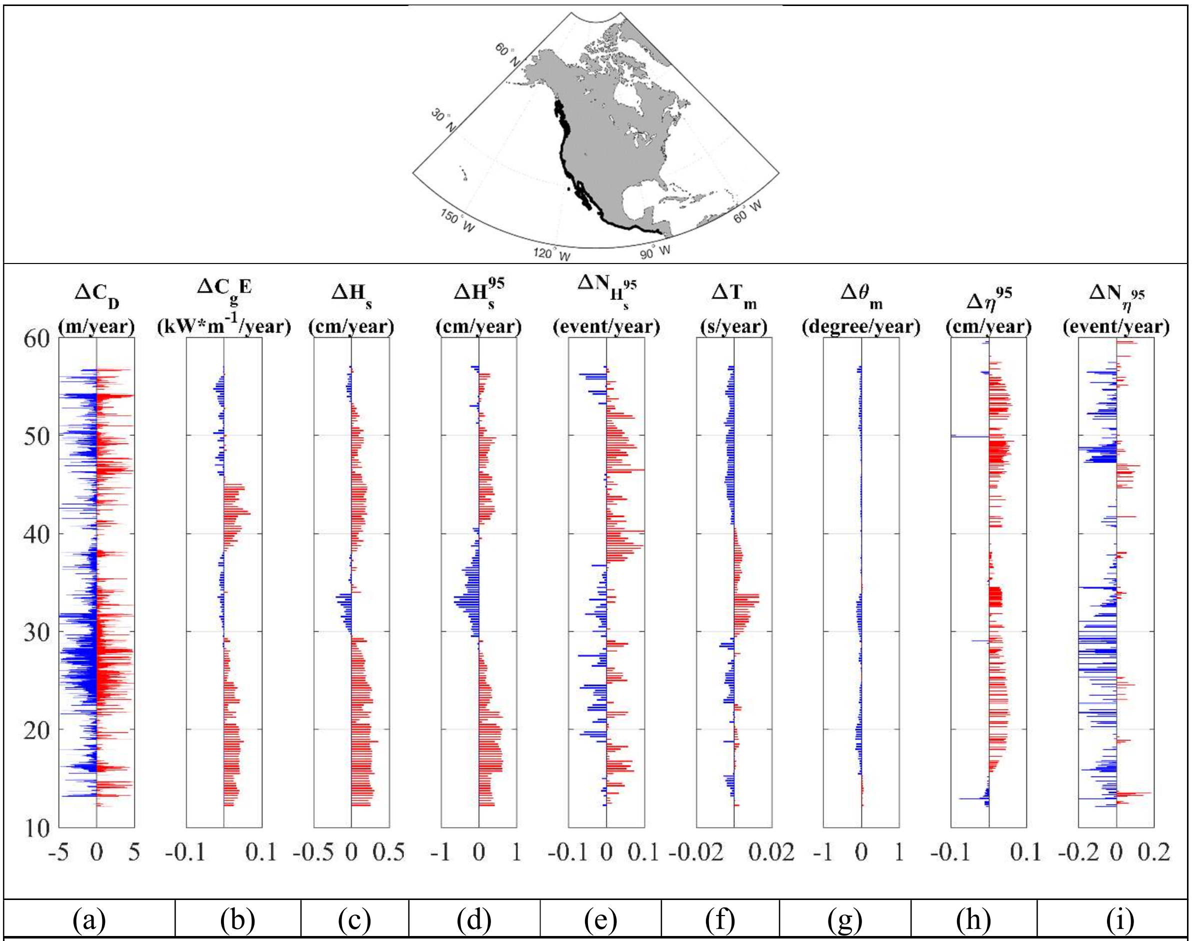

Figure S12. Latitudinal distribution of: (a) shoreline recession/progradation, ΔC_D for sandy beaches (red is progradation and blue is recession), (b) trend in wave energy flux ($\Delta C_g E$), (c) trend in significant wave height (ΔH_s), (d) trend in 95th percentile significant wave height (ΔH_s^{95}), (e) trend in number of extreme wave events above the 95th percentile ($\Delta N_{H_s^{95}}$), (f) trend in mean wave period (ΔT_m), (g) trend in mean wave direction ($\Delta \theta_m$), (h) trend in magnitude of storm surge ($\Delta \eta^{95}$) and (i) trend in number of storm surges events ($\Delta N_{\eta 95}$) for the west coast of North America. Increasing trends shown in red and decreasing trend in blue. Wave parameters are from the Liu et al. dataset. The insert at the top shows the continent of North America highlighting the coastline region considered.

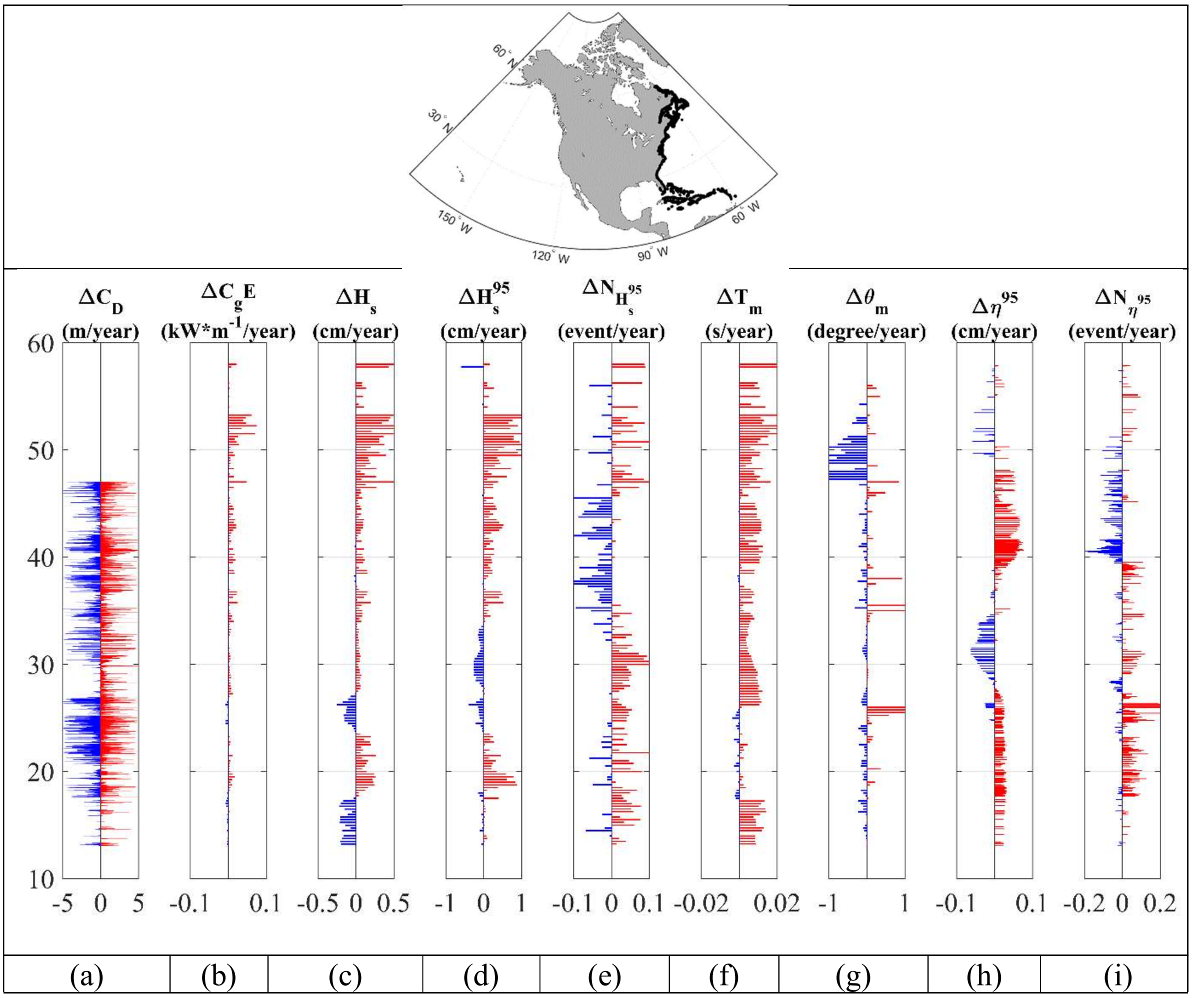

Figure S13. As for Figure S12 but for the east coast of North America.

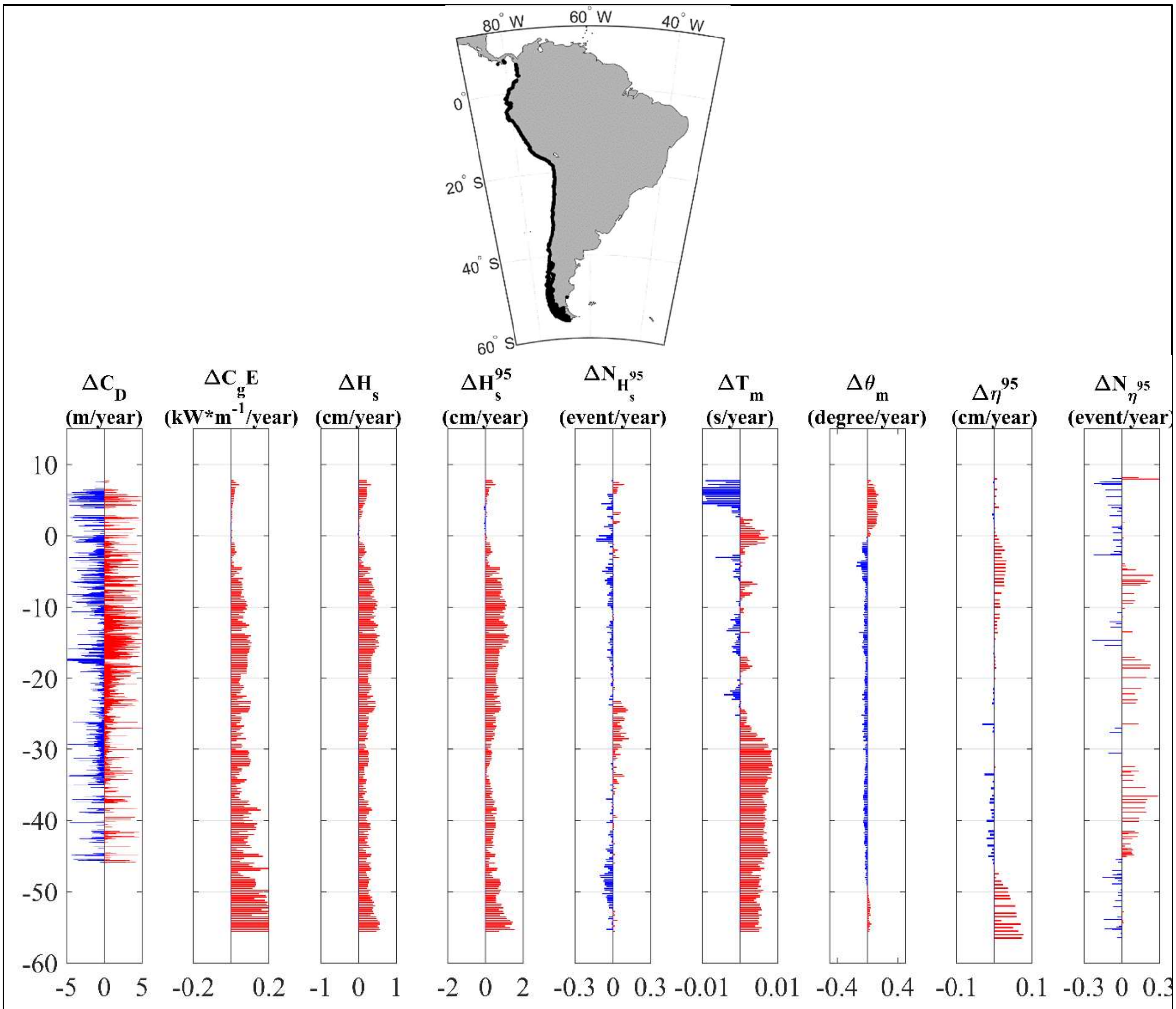

(a)	(b)	(c)	(d)	(e)	(f)	(g)	(h)	(i)

Figure S14. As for Figure S12 but for the west coast of South America.

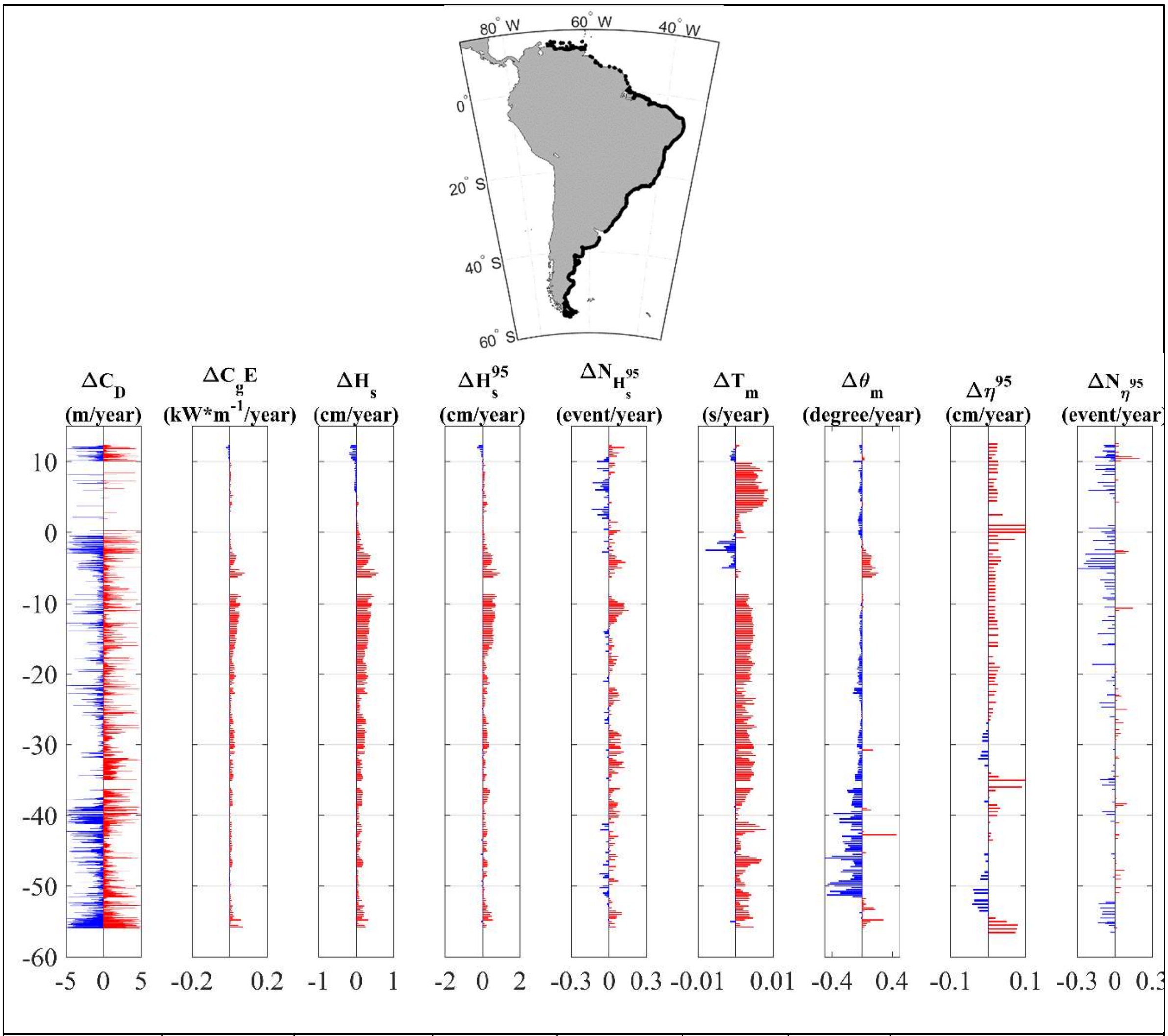

(a)	(b)	(c)	(d)	(e)	(f)	(g)	(h)	(i)

Figure 15. As for Figure S12 but for the east coast of South America

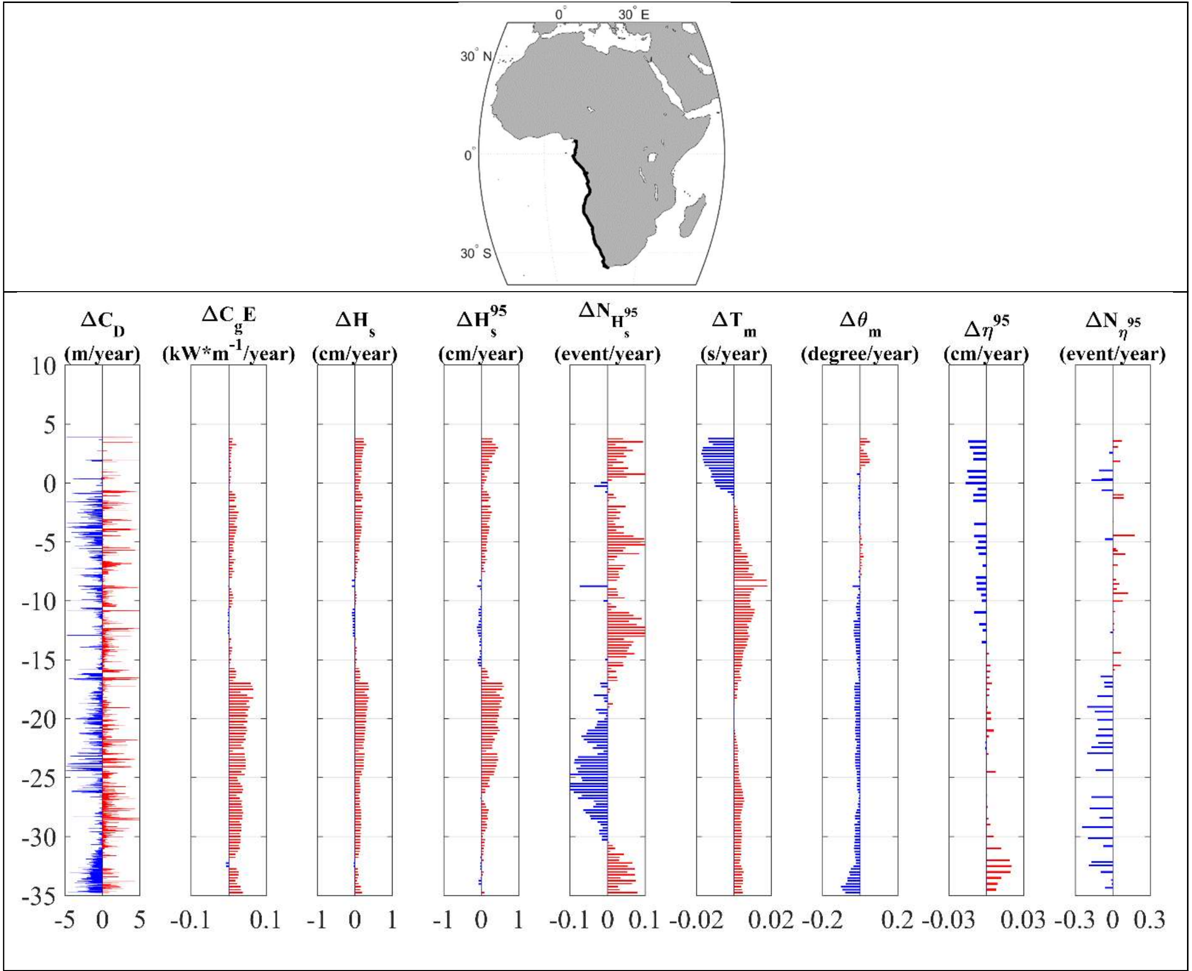

(a)	(b)	(c)	(d)	(e)	(f)	(g)	(h)	(i)

Figure S16. As for Figure S12 but for the west coast of Africa.

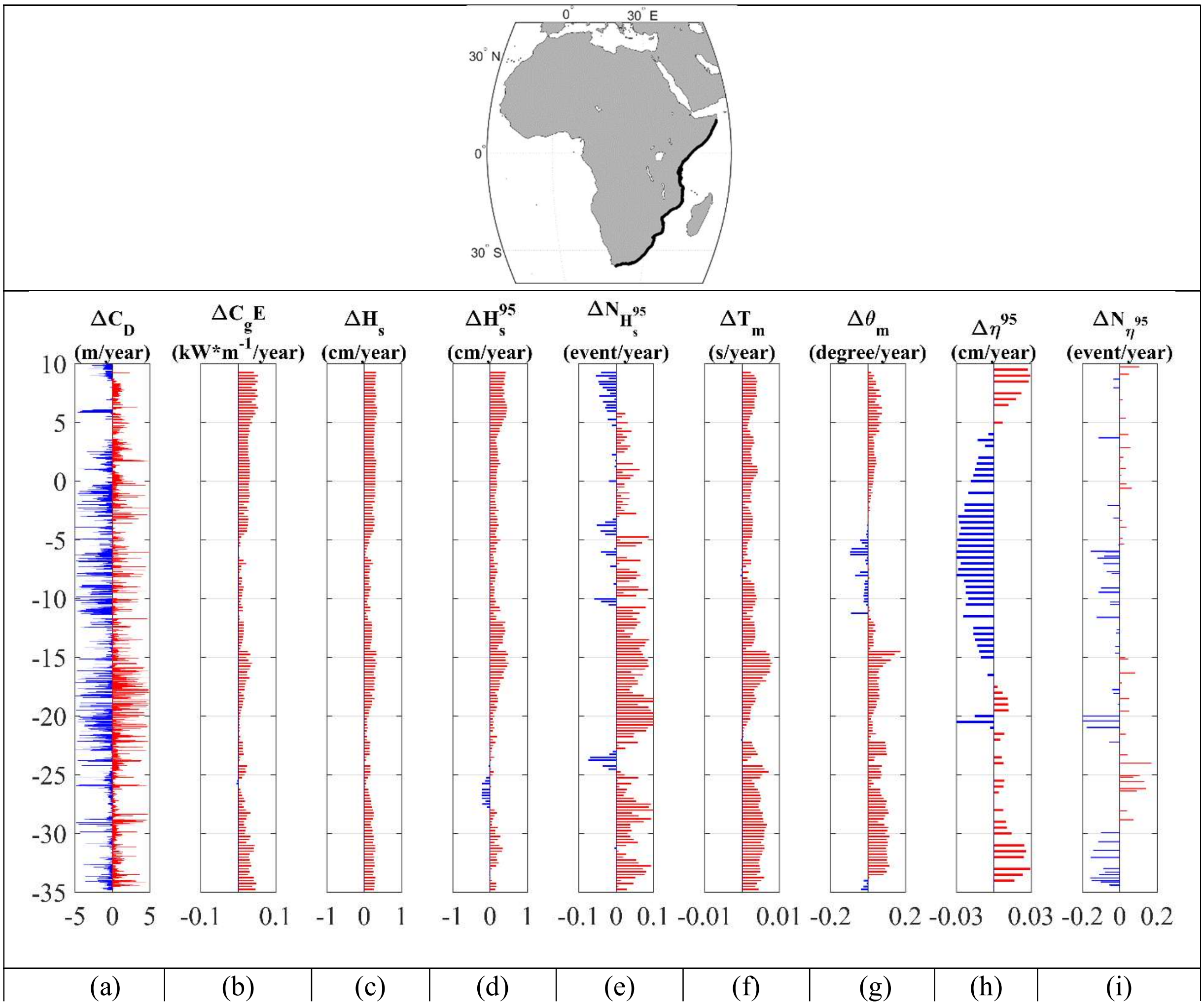

Figure S17. As for Figure S12 but for the east coast of Africa.

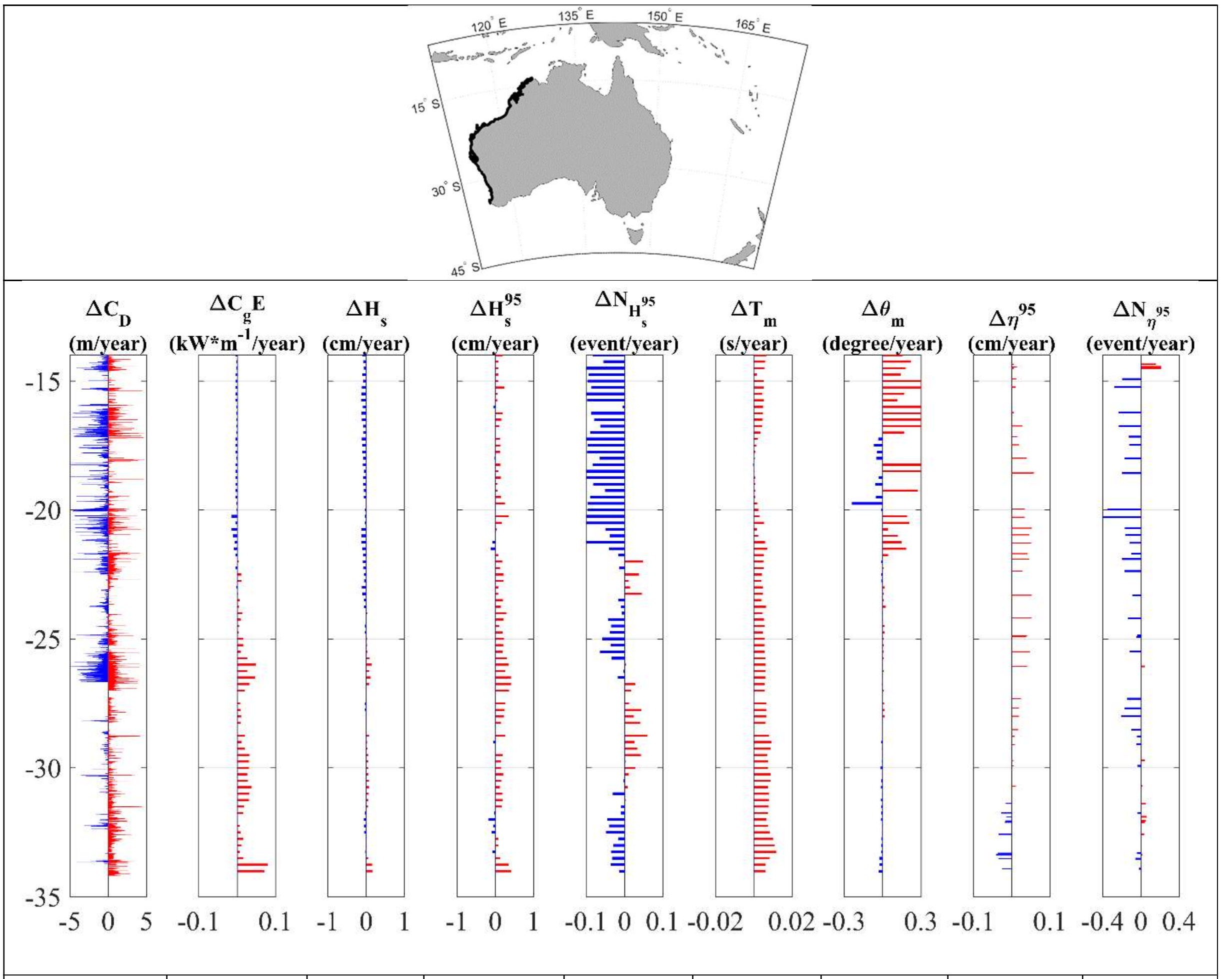

(a)	(b)	(c)	(d)	(e)	(f)	(g)	(h)	(i)

Figure S18. As for Figure S12 but for the west coast of Australia.

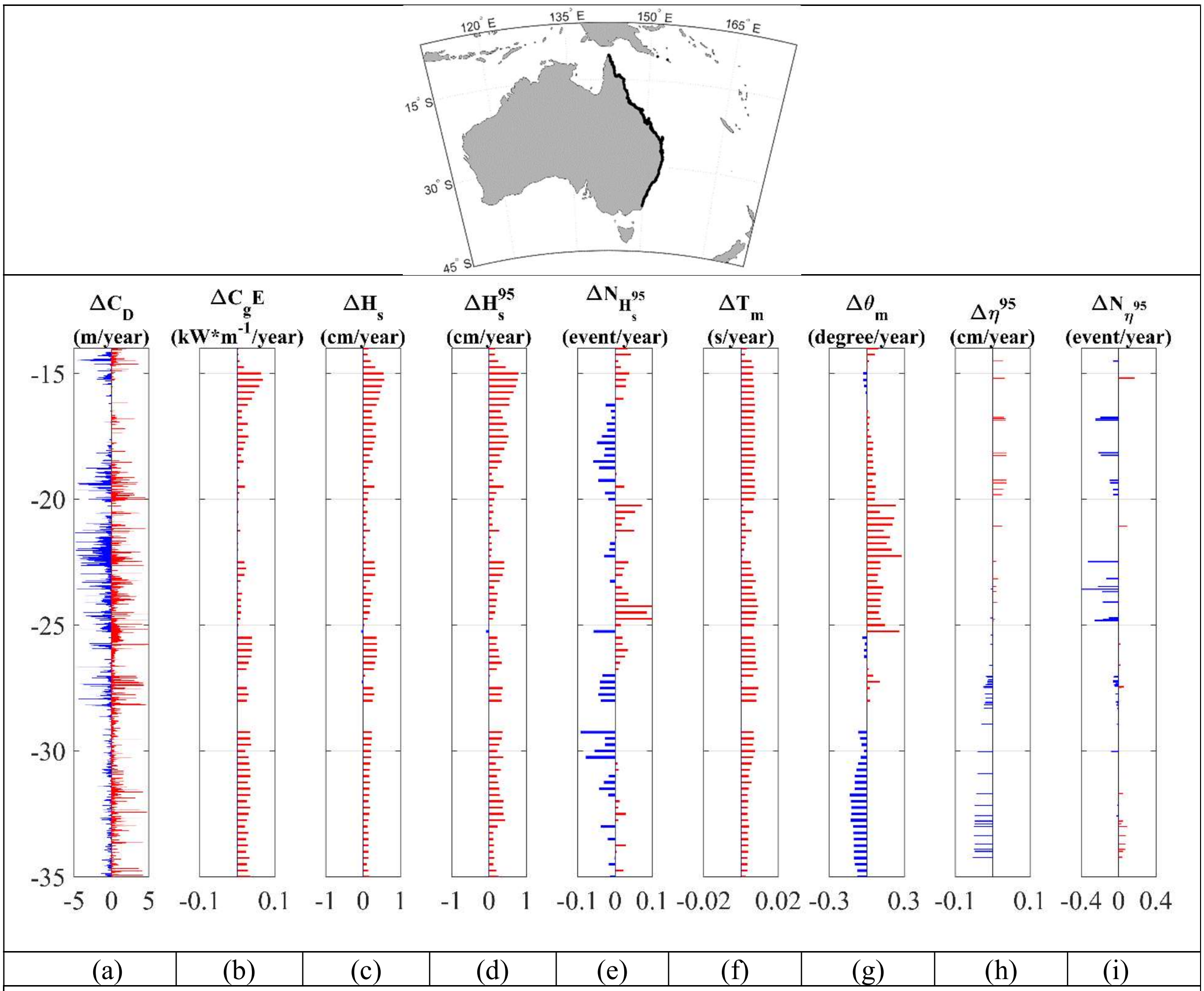

Figure S19. As for Figure S12 but for the east coast of Australia.

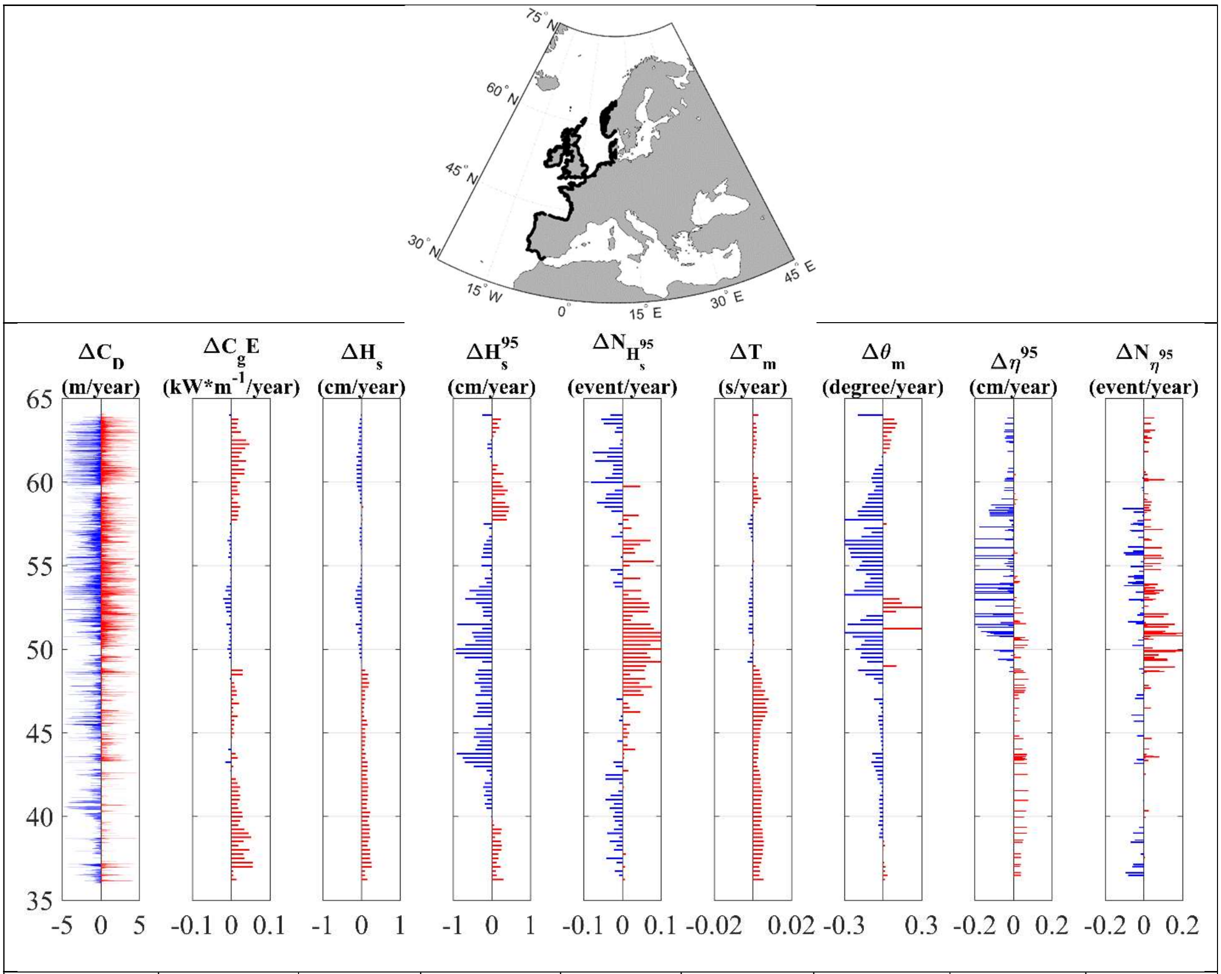

(a)	(b)	(c)	(d)	(e)	(f)	(g)	(h)	(i)

Figure S20. As for Figure S12 but for the west coast of Europe.

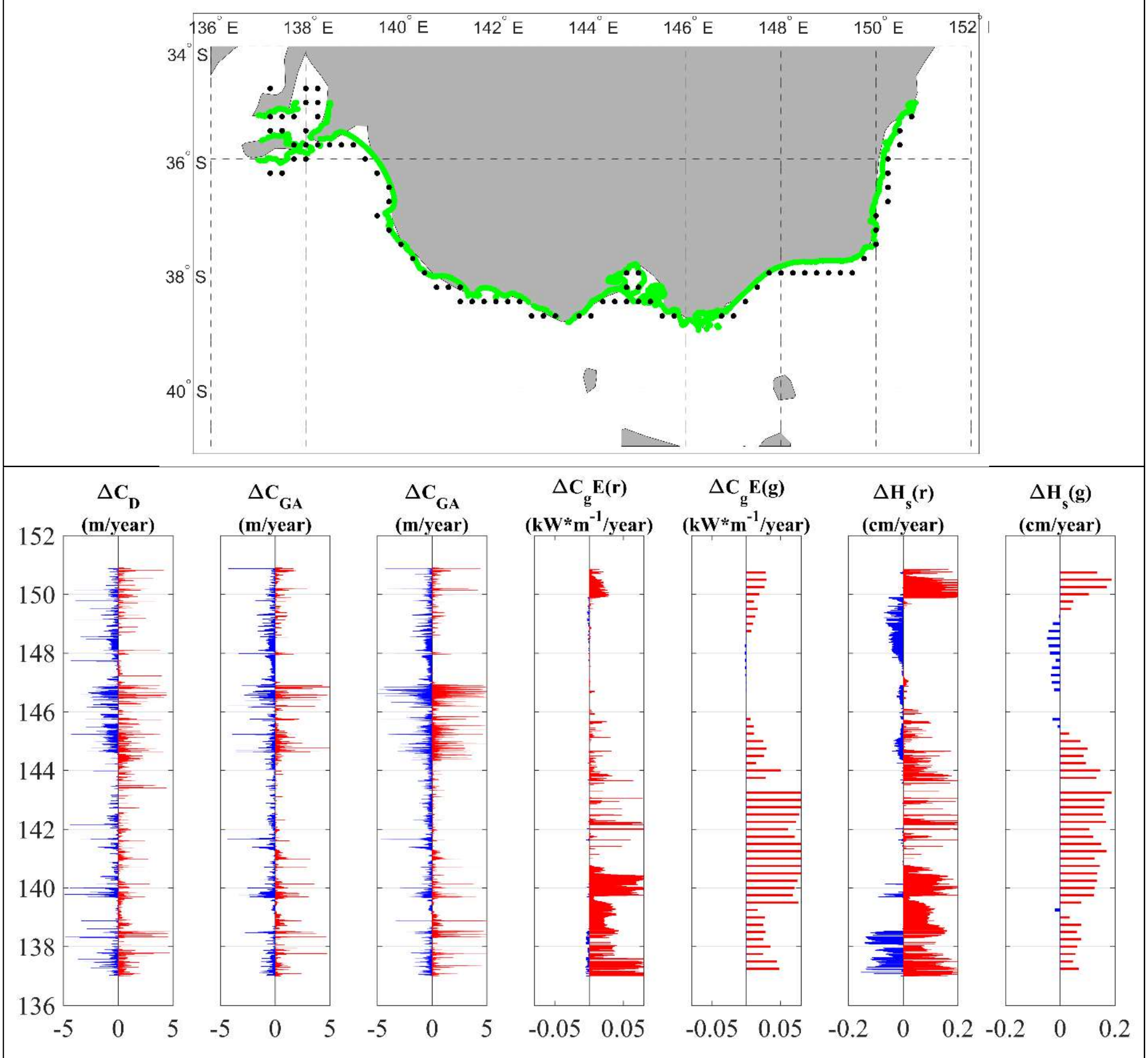

(a)	(b)	(c)	(d)	(e)	(f)	(g)

Figure S21. Longitudinal distribution of: (a) shoreline recession/progradation from the *Delft* (ΔC_D) sandy beach dataset, (b) shoreline erosion/accretion from the *Geoscience Australia* (ΔC_{GA}) dataset for sandy beaches, (c) shoreline recession/progradation from the *Geoscience Australia* (ΔC_{GA}) all beaches, red color shows progradation and blue is recession. (d) Trend of annual mean values of wave energy flux from the Liu et al. regional ($\Delta C_g E(r)$) dataset, (e) trend of wave energy flux from the Liu et al. global ($\Delta C_g E(g)$) dataset, (f) trend in significant wave height from Liu et al. regional ($\Delta H_s(r)$) and (g) trend in significant wave height from Liu Q. et al. global ($\Delta H_s(g)$) dataset. Positive values shown in red and negative values in blue. The insert at the top shows the location in Victoria and coastline of Australia. The island of Tasmania is to the south. Green points are data locations for the regional dataset and black points for the much coarser global dataset.
